# Effect of a patient-driven perioperative intervention on health literacy: A stepped-wedge cluster randomised sub-study

**DOI:** 10.1371/journal.pone.0352245

**Published:** 2026-06-24

**Authors:** Ann Kristin Sandsbakk Austarheim, Kristin Harris, Hilde Valen Wæhle, Anette Storesund, Randi Julie Tangvik, Arvid Steinar Haugen

**Affiliations:** 1 Department of Anaesthesia and Intensive Care, Haukeland University Hospital, Bergen, Norway; 2 Department of Nursing and Health Promotion, Acute and Critical Illness, Faculty of Health Sciences, Oslo Metropolitan University, Oslo, Norway; 3 Department of Health and Caring Sciences, Western Norway University of Applied Sciences, Bergen, Norway; 4 Department of Global Public Health and Primary Care, University of Bergen, Bergen, Norway; 5 Department of Research and Development, Haukeland University Hospital, Bergen, Norway; 6 Department of Clinical Medicine, University of Bergen, Bergen, Norway; Tarbiat Modares University Faculty of Medical Sciences, IRAN, ISLAMIC REPUBLIC OF

## Abstract

We hypothesised that a patient-driven safety checklist would enhance patients’ health literacy. This study was conducted as a sub-study of a multicentre cluster trial with a stepped wedge design. Data were collected between March 2022 and February 2024. Healthcare personnel were partially blinded to group allocation and fully blinded to the health literacy questionnaire outcome. The study included seven surgical specialties (clusters) from a tertiary teaching hospital and two community hospitals in Norway. The patients were included from a pool within the cluster trial. In each of these seven clusters, 50 patients were randomly selected from 100 eligible patients both the control and the intervention group using a computer-generated randomisation procedure. This resulted in a total sample of 700 patients: 350 in each group, response rate 49.3%. Adults (≥18 years) undergoing elective surgery, fluent in Norwegian, living at home, and without cognitive impairment were included. Standard surgical information combined with a patient safety checklist consisting of items with instructions and information (medication safety, preparations, activity restrictions, complications, and follow-ups). The checklist was delivered preoperatively (≤ eight weeks). Controls received standard surgical information. No significant differences were observed in mean scores across the nine health literacy questionnaire outcome domains between the control and intervention groups. In this stepped wedge cluster trial, a preoperative patient safety checklist did not improve health literacy scores compared to standard care. Nevertheless, an interaction effect with time for the domain “Actively managing my health”, may indicate that the patient-driven checklist could support elective surgical patients’ health management. This finding aligns with qualitative feedback and warrants further investigation using larger samples and more sensitive and context-specific measurement tools. **Trial registration:** Registration date in the ClinicalTrials.gov, 20 March 2017, ID: NCT03105713.

## Introduction

Health literacy is a critical component enabling patients to participate effectively in surgical care, including informed decision-making and adherence to perioperative instructions [1,2]. However, evidence suggests that low health literacy is prevalent among surgical patients [3, 4]. Surgical pathways are characterized by high complexity and substantial information demands across the surgical trajectory [5]. Low health literacy has been associated with an increased risk of poor medical comprehension, surgical complications including surgical site infections and prolonged hospital stays [5–8]. Such outcomes may be explained by misunderstandings of preoperative information and discharge instructions [6]. Given this established association, further prevalence studies of health literacy within surgical populations may be of limited practical value [4]. Instead, efforts should be directed toward the development and implementation of interventions aimed at strengthening patients’ health literacy [4]. Despite this need, a recent literature review reveals a scarcity of health literacy interventions tailored to surgical patients [9]. It may especially be challenging to measure and reliably interpret outcome in patients undergoing surgical interventions due to heterogeneity in patient and procedures characteristics. This partly reflects the limited knowledge and understanding of health literacy as a concept within surgical context [9].

A novel Patient Safety Checklist (PASC) has been developed to engage elective surgical patients in optimising their health and safety throughout the surgical pathway [10]. The PASC provides structured items with information, instructions, and safety advices both preoperatively and before discharge from hospital, promoting interactions with healthcare professionals [10]. A concrete PASC item is: “Do you use blood-thinning medication?” The item is followed by instructions regarding medication management and advice to contact the surgical ward if clarification is needed. Such items are designed to support patients in identifying and acting upon important perioperative safety issues [10]. Results from previous PASC-studies indicates that PASC users feel more in control as surgical patients, have a clearer understanding of actions to prevent complications, recognises important information, and are able to screen themselves for the risk of malnutrition [10–12]. All these features align with health literacy principles [13]. However, PASC’s impact on surgical patients’ health literacy has not been formally evaluated. Hence, the current study was conducted as a sub-study within a stepped wedge cluster randomized controlled trial to address this knowledge gap. This study assessed all nine distinct domains of health literacy as defined by the Health Literacy Questionnaire (HLQ) providing a comprehensive evaluation of patients’ health literacy skills and experiences [14]. It was hypothesised that the patient-driven safety checklist would enhance surgical patients’ health literacy.

## Materials and methods

### Study design and participants

**Fig 1:**
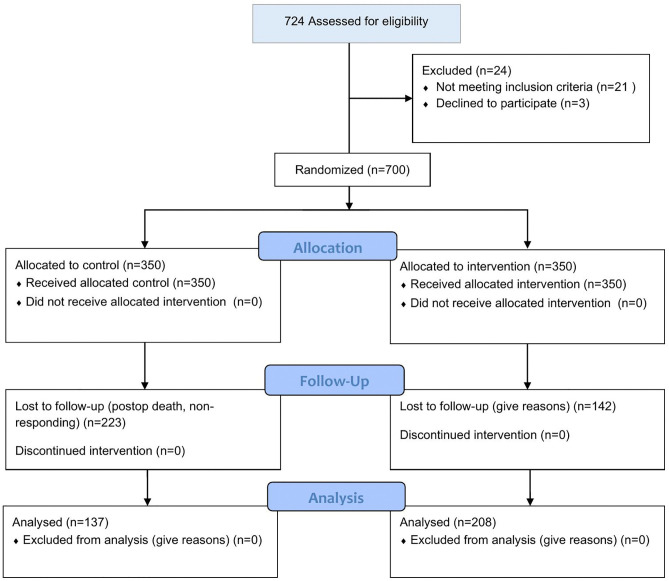
Consort 2010 Flow Diagram. This flow diagram illustrates the process of participant recruitment, allocation, follow-up, and analysis in the study.

This sub-study followed the control and intervention phases of a larger multicentre stepped-wedge cluster randomised controlled trial. Data were collected between 1 March 2022 and 29 February 2024. The study sample included elective surgical patients from a tertiary teaching hospital and two community hospitals in Western Norway [15]. Eligible participants underwent surgery within seven surgical specialties (clusters): Orthopaedic, ear-nose-throat/maxillo-facial, neurosurgery, breast-endocrinology, cardio-thoracic, gastrointestinal, or general surgery [15]. Randomisation of clusters and patients was concealed from the clusters until they received the intervention. In the control group all clusters performed standard surgical care. Following a randomised order the first cluster then switched to intervention group where patients used the PASC intervention. The other clusters still providing standard surgical care. After the next two months, the second cluster switched to intervention group, and this went on until all clusters had crossed over from control to intervention groups. For the sub-study this included a 14-months period. Information about the study and consent form were sent by mail up to eight weeks before hospital admission. The HLQ was sent to participants up to three months after discharge, and one reminder was sent to non-responders in accordance with ethical board permission. The patients were included from a pool within the cluster trial. In each of these seven clusters, 50 patients were randomly selected from 100 eligible both the control and the intervention group using a computer-generated randomisation procedure (RANDOM.ORG). This resulted in a total sample of 700 patients: 350 in the control groups and 350 in the intervention groups. This random sub-sampling approach was primarily done to minimize unnecessary patient burden and exposure to research questionnaires, and secondly for logistical and resource constraints. Fig 1 presents the participant selection and randomisation process.

Inclusion criteria were elective surgery, age ≥ 18 years, fluent in Norwegian, residence outside institutions (e.g., nursing homes). Absence of cognitive impairment was assessed collaboratively with ward nurses in each cluster based on patient condition and medical records. This study followed the updated reporting guidelines for randomised trials CONSORT 2025 (S1 File) [16]. The study protocol was approved by the Regional Committee for Medical and Health Research Ethics in Western Norway (REC West, 2016/1102), and by the hospitals’ managers. In this sub-study of the larger trial written informed consent was obtained from all participants in the intervention group prior to trial participation, including consent for the publication of anonymised data. Participants in the control group signed written informed consent along with the reponses to the questionnaire. All authors had access to data containing information that could potentially identify individual participants during and after the data collection period.

The study was conducted in accordance with the Declaration of Helsinki [17], and the study protocol is available in the S2 File.

### PASC intervention

Patients in both the control and intervention groups received standard surgical information. In addition, patients in the intervention group received the PASC checklist and were invited to use it [18]. The checklist tool was developed and validated in a collaboration between safety researchers, healthcare personnel and surgical patients, and has been published elsewhere [10]. The preoperative section had 26 checklist items and 3 advisory items that covered medication safety, health optimization, discharge planning, and preparatory instructions, encouraging patients to discuss missing information with their healthcare providers [18]. The postoperative section had 22 items addressing potential complications, activity restrictions, medication safety, and follow-up instructions [18]. The checklist items were accompanied by instructions encouraging patients to ask or request direct health and safety-related questions from the healthcare personnel involved in their surgery [10,11]. PASC was provided in paper form and digitally via the national health platform up to eight weeks before surgery by trained researchers with clinical backgrounds within anaesthesia and intensive care nursing. Completed checklists were returned before discharge, either by mail or digitally. No adverse events related to the intervention were observed. Interim analyses were not performed during this sub-study, though it followed the stepped wedge design of the clinical trial.

A stepped wedge cluster randomised design was used to randomise the clusters for when they shifted from control to intervention steps. This method is recognised as robust for evaluating patient safety and health service interventions while minimising contamination bias [19]. The personnel implementing the enrollment and assigned participants to the intervention had access to the random allocation sequence. Healthcare personnel working within the clusters were fully blinded to the study outcome and partially blinded for participant allocation to the checklist. Healthcare personnel in operating theaters and post anaesthesia care units, were fully blinded to the study outcome and to patients’ use of the checklist. The research assistant plotting data was blinded to group allocation.

### Primary outcome

The primary outcome in this study was the HLQ domains [14]. Although the HLQ has demonstrated good psychometric properties across diverse populations [14,20], there remains a theoretical possibility of ceiling effects. Such effects have been reported in other HLQ studies [21,22]. Given the limited existing evidence on possible ceiling effects of the HLQ in his specific population, this aspect needs to be analysed. To assess patients experiences the validated self-reported HLQ was utilised [14,20]. HLQ covered nine conceptually distinct domains of health literacy, making it suitable for intervention evaluations due its validity-driven approach [14]. Domains: 1. Feeling understood and supported by healthcare providers, 2. Having sufficient information to manage my health, 3. Actively managing my health, 4. Social support for health, 5. Appraisal of health information, 6. Ability to actively engage with healthcare providers, 7. Navigating the healthcare system, 8. Ability to find good health information, and 9. Understanding health information well enough to know what to do. Each domain consisted of four to six questions, 44 questions in total [14,20]. The response scale for the first five domains were scored as: 1 = strongly disagree, 2 = disagree, 3 = agree, 4 = strongly agree. The domains six to nine were scored as 1 = cannot do or always difficult, 2 = usually difficult, 3 = sometimes difficult, 4 = usually easy, and 5 = always easy. A license was provided for using HLQ in this project (L210731S).

### Sample size

The required number of participants was determined through a sample size analysis. To detect a clinical meaningful mean group difference of 0.30 between the two groups, a minimum of 103 participants were needed in each group of the PASC trial according to the Statistical Analysis Plan [15]. A minimum response rate was estimated to be approximately 30%. This assumption was based on previous research within the larger hospital [23]. The target sample for the data collection was set at 350 participants in both the control and the intervention groups, 700 in total [15].

### Statistical analyses

Descriptive statistics with frequencies, percentages, means, and standard deviations were used to assess patient characteristics. Chi-squared test and independent t-test were employed to identify any differences between the control and intervention groups, as appropriate [15]. We analysed the frequencies of HLQ items across the nine domains to describe the distribution of responses. Analysis of Variance (ANOVA) was used to compare mean score differences between control and intervention groups on the HLQ domains. Further, we used General Linear Mixed Model to assess the impact PASC had on the nine HLQ domains. Each HLQ domain was treated as dependent variables, with control/intervention and time as fixed factors. The clusters were modeled as random effects to produce valid inferences for the mixed-effects model, with patient characteristics were treated as covariates. Additionally, we investigated if the outcomes were influenced by interactions between control/intervention and time by adding this interaction to the factor model. Floor and ceiling effects were analysed to assess the distribution of the lowest and highest item scores across each HLQ domain. Floor/ceiling effects will be considered present if >15% of participants achieved the minimum or maximum score [24]. Patterns of missing data were controlled for patient characteristics and the HLQ domain items. Baseline characteristics of responders and non‑responders were compared to assess potential attrition biases. Responders and non-responder differences were examined for the variables age, sex, comorbidities (ASA), and clusters by using descriptive analyses. To avoid risk of losing power in the analyses, the regression analysis was performed with imputed data for education, civil status, work status, and the HLQ domains. Imputation was generated by 10 imputations with an automatically imputation method based on the missing pattern in the data. For sensitivity analysis we imputed non-responders’ missing outcome values based on the observed responder outcomes. Internal consistency was assessed using Cronbach´s alpha, with a cut-off set to > 0.7 considered adequate [25]. To account for variability in Cronbach’s alpha domain measures, we calculated 95% confidence intervals (CI’s) using the intraclass correlation coefficient (ICC) with absolute agreement, and the average measures. All analyses were performed in SPSS 31.0 (IBM Corp, Armonk, NY), with two-sided P-value ≤0.05 considered statistically significant.

## Results

### Patient characteristics

A total of 49.3% (345/700) patients completed the HLQ (mean [SD] age, 62.0 [12.9] years; 183 [53.0] female and 162 [47.0] male), including 137 in the control group and 208 in the intervention group. There were no statistically significant differences in patient characteristics (Table 1).

**Table 1:** Characteristics of the participants.

	Participants, n (%)			
	Total (n = 345)	Control Group (n = 137)	Intervention roup (n = 208)	*P* Value ^a^
**Age**, mean (SD)	62.0 (12.9)	62.65 (13.1)	61.49 (12.8)	0.42
**Sex**				0.32
Female	183 (53.0)	68 (49.6)	115 (55.3)	
Male	162 (47.0)	69 (50.4)	93 (44.7)	
**ASA**				0.62
ASA I	44 (12.8)	17 (12.4)	27 (13.0)	
ASA II	210 (60.9)	83 (60.6)	127 (61.1)	
ASA III	83 (24.1)	32 (23.4)	51 (24.5)	
ASA IV	8 (2.2)	5 (3.6)	3 (1.4)	
Missing	–	–	–	
**Civil status**				0.33
Married	220 (63.8)	90 (68.7)	130 (62.8)	
Partner/cohabitant	71 (20.6)	22 (16.8)	49 (23.7)	
Single	47 (13.6)	19 (14.5)	28 (13.5)	
Missing	7	6	1	
**Education**				0.26
Primary school	31(9.0)	9 (7.5)	22 (10.7)	
Upper secondary	136 (39.4)	57 (47.5)	79 (38.5)	
University/College	158 (45.8)	54 (45.0)	104 (50.7)	
Missing	20	17	3	
**Work status**				0.56
Work full time	133 (38.6)	48 (35.8)	85 (41.1)	
Work part time	22 (6.4)	8 (6.0)	14 (6.8)	
Not employed	186 (53.9)	78 (58.2)	108 (52.2)	
Missing	4	3	1	
**Surgical specialties**				0.19
Orthopaedic	45 (13.0)	18 (13.1)	27 (13.0)	
ENT/ maxillo-fascial	32 (9.3)	6 (4.4)	26 (12.5)	
Cardio-thoracic	55 (15.9)	21 (15.3)	34 (16.3)	
Neurosurgery	50 (14.5)	24 (17.5)	26 (12.5)	
Breast- and endocrine	55 (15.9)	20 (14.6)	35 (16.8)	
Gastrointestinal	59 (17.1)	27 (19.7)	32 (15.4)	
General surgery	49 (14.2)	21 (15.3)	28 (13.5)	

Abbreviations: SD = Standard Deviation; ASA = American Society of Anaesthesiology risk assessment score, ENT = Ear, nose, and throat

^a^Independent samples t-test and chi-squared test were utilised to compare the two groups.

### Health literacy domain outcomes

The distribution of responses for HLQ items varied across the domains. Frequencies for items in domains 1–5 are shown in S1 Table, while frequencies for domains 6–9 are presented in S2 Table. Mean scores on all nine HLQ domains between the control and intervention groups, were not statistically significant different (P > .05) and had very small effect sizes (eta squared = 0.001–0.003). Mean scores ranged from 2.67 to 3.10 for domains 1–5 and 3.51 to 3.85 for domains 6–9 (Table 2).

**Table 2:** Participants’ mean scores on the Health Literacy Questionnaire domains.

	Control			Intervention				
Health Literacy Questionnaire domains								Eta Squared
	n	Mean	SD ^a^	n	Mean	SD	*P* Value ^a^	
1. Feeling understood and supported by healthcare providers	137	3.1	0.56	208	3.05	0.64	0.429	0.002
2. Having sufficient information to manage my health	136	3.03	0.47	208	2.97	0.52	0.308	0.003
3. Actively managing my health	136	3.03	0.5	208	3.06	0.47	0.634	0.001
4. Social support for health	137	3.1	0.49	208	3.08	0.53	0.648	0.001
5. Appraisal of health information	136	2.67	0.55	208	2.67	0.56	0.98	<0.001
6. Ability to actively engage with healthcare providers	137	3.81	0.61	207	3.77	0.66	0.5	0.001
7. Navigating the healthcare system	136	3.55	0.61	207	3.51	0.67	0.599	0.001
8. Ability to find good health information	137	3.59	0.56	207	3.56	0.62	0.72	<0.001
9. Understanding health information well enough to know what to do	137	3.8	0.57	207	3.85	0.54	0.45	0.002

Abbreviations: SD = Standard Deviation.

^a^*P* Value represent comparison of the Health Literacy Questionnaire domains with Analysis of Variance (ANOVA).

Overall results from the mixed-effects regression analysis showed no statistically significant differences between the groups (Table 3). In the mixed-effects model with interaction between control/intervention and time, pairwise comparisons suggested a significant positive mean difference for the intervention at 0.28, 95% CI (−0.54, −0.2) with P value 0.037 for domain 3 (Actively managing my health). A total of 51.4% of the study sample were non-responders. Healthier, younger patients were less likely to respond, with mean age 54.1 vs. 62.0 years; P = 0.025, and ASA I classification at 60.4% vs. 39.6%. No sex differences were observed (P = 0.762). Sensitivity analysis with imputed data including non-responders showed very small changes and indicated robustness of the analyses (S3 Table). Missing data were minimal for the domains. Across both groups, 2.3% of data were missing for marital status, 5.8% for education level, and 1.2% for work status. The Cronbach’s alpha values ranged from 0.725 to 0.873 across the nine domains and are reported with corresponding 95% CI’s in the S4 Table.

**Table 3:** Mixed-effect model analysis of PASC’s impact on the Health Literacy Questionnaire domains.

Health Literacy Questionnaire domains	Control/Intervention ß^a^	95% CI	*P* Value
1: Feeling understood and supported by healthcare providers	−0.14	(−0.49-0.21)	0.433
2: Having sufficient information to manage my health	−0.12	(−0.45-0.20)	0.462
3: Actively managing my health	−0.13	(−0.44-0.19)	0.440
4: Social support for health	0.04	(−0.28-0.37)	0.802
5: Appraisal of health information	−0.10	(−0.40-0.20)	0.522
6: Ability to actively engage with healthcare providers	−0.03	(−0.38–0.31)	0.854
7: Navigating the healthcare system	0.04	(−0.32-0.40)	0.836
8: Ability to find good health information	−0.05	(−0.40-0.30)	0.791
9: Understanding health information well enough to know what to do	−0.05	(−0.37-0.27)	0.763

Abbreviations: PASC = Patient Safety Checklist; CI = Confidence Interval ^a^ ß = beta representing mean difference from intercept, with intervention group as reference. Control/intervention, time, and interaction were fixed factors, with clusters as random effects. The model was adjusted for age, sex, American Society of Anaesthesiology comorbidity risk classification scores (ASA), civil status, education, work status. Analysis was performed with missing imputation for education, work and civil status and the domains.

## Discussion

To our knowledge, this is the first study in a stepped wedge trial to evaluate the impact of a patient safety checklist combined with standard surgical information on elective surgical patients’ health literacy using the HLQ. The intervention was not associated with statistically significant changes in HLQ domain scores, and the overall study hypothesis was therefore not supported. The mixed-effects model with including cluster as random effects, adjusting for relevant covariates did not alter these findings. However, pairwise comparisons suggested that domain 3 (Actively managing my health) was significantly improved, when accounting for interaction between time and control/intervention groups. This finding may indicate that a larger sample could identify possible impact of the patient-driven checklist. This was supported by findings in a recent published qualitative study, where using the PASC raised their awareness on safety issues, and led to actions for ensuring medication control, optimisation of health, and increasing their interactions with healthcare personnel [11]. Rather than indicating a lack of relevance of the intervention per se, the overall results are best interpreted considering methodological and contextual factors. In particular, the high baseline health literacy observed in this Norwegian cohort, together with ceiling effects in several HLQ domains, suggests that the instrument may have had limited sensitivity to detect modest or context‑specific changes attributable to the PASC intervention.

PASC is considered highly operational, context specific and oriented toward patient safety, focusing on preparation for elective surgical patients and supporting recovery planning following hospital discharge [10,11,18]. The core elements of PASC align closely within the dimensions of the HLQ [10,14]. In a wider perspective, the HLQ items assess broader underlying health literacy structures [14], and may be less sensitive to changes in this patient population. For instance, two domains “Feeling understood and supported by healthcare providers” (domain 1) and “Social support for health” (domain 4), primarily reflect existing relationships with family or providers, such as having a trusted general practitioner or relative to rely on [14]. While these elements are conceptually relevant to PASC, the checklist cannot directly change family structures or preexisting interpersonal relationships with healthcare personnel. Its function is rather to prompt patients to use these preexisting relationships more effectively. This distinction could explain why no change was measured in these domains despite patients’ reported engagement with PASC [11].

Another plausible explanation of the findings relates to the high baseline HLQ scores observed in the control group. Mean scores indicated moderate to high levels of health literacy, suggesting that many participants already possessed strong competencies prior the intervention. As reported in a previous study, HLQ item scores frequently clustered toward the higher end of the item scales [21]. In the present study,  > 15% of participants scored the maximum score on items in six of the nine HLQ domains (strongly agree or always easy), indicating ceiling effects [24]. This assumption was consistent with findings observed in the Dutch HLQ validation, where ceiling effects also were observed in six of nine domains observed [21]. Ceiling effects are not unique for the HLQ. Other health literacy instruments, such as the Test of Functional Health Literacy in Adults (TOFHLA) and the European Health Literacy Survey (HLS-EU-Q47), have shown similar limitations [22]. Conversely, the Newest Vital Sign (NVS) demonstrated floor effects, limiting its ability to distinguish among individuals with low literacy [22].

In a previously mentioned qualitative study of PASC, patients experienced the checklist as a structured and useful tool that increased their awareness of important information, strengthened their preparedness, and prompted concrete safety-related actions [11]. These experiences suggest shifts in understanding, control, and readiness to act; dimensions closely related to health literacy but more situational and practice-oriented than the broader constructs captured by the HLQ. Thus, qualitative findings indicate that aspects of patient engagement not readily detected by fixed-response measurements, underscoring the value of combining quantitative and qualitative approaches when evaluating complex interventions [26]. Qualitative findings from the Optimizing Health Literacy and Access (Ophelia) project revealed meaningful health literacy improvements which were not reflected in their quantitative data [26]. These observations support the idea that qualitative research may precede and complement measurable data, particularly when studying complex constructs as health literacy [26,27].

Although no immediate changes in HLQ domain scores were observed following completion of the intervention, PASC was utilised by patients across their surgical pathways, with the intervention initiated prior to hospital admission [10]. Potential long-term effects on patients’ health literacy are uncertain. In a randomised controlled study, a follow-up communication intervention study among kidney transplant recipients improvements were found in HLQ scores after one year: “Appraisal of health information” (domain 5) and “Navigating the healthcare system” (domain 7) [28]. Nonetheless, only the latter remained significant and even stronger after two years [29]. This suggests that health literacy may vary over time and evolve with patients’ experiences at different stages of care.

### Limitations

Several limitations should be considered when interpreting the results. The overall health literacy domain levels demonstrated ceiling effects in both control and intervention groups, which may have limited the ability to detect changes. Even though a priory sample size was estimated, it is possible that a higher number of respondents and participants could have increased the study’s overall ability to detect changes. Especially since younger and healthier participants responded less than older patients. In the general Norwegian population, younger people between 18–24 years are reported to have challenges with interacting with healthcare professionals [30]. Based on this underlying difference between responders and non-responders, we cannot exclude the risk that the statistical power was limited and could have increased with a larger sample. However, even if sensitivity analysis based on responder outcomes could not completely account for the large proportion of non-responses, it showed that with imputed data for non-responders there were no significant changes, indicating robustness in the analysis.

Another possible limitation is the inclusion criteria of the larger clinical trial. Patients with higher risk of low health literacy, including individuals with limited education, migration backgrounds, multiple chronic conditions, or social isolation [31], may have been underrepresented in this study. Patients with low health literacy may also be less likely to complete lengthy surveys [32], which could further reduce sample variability and limit the ability to detect PASC’s impact on patients’ HLQ domain responses. Nonetheless, the HLQ captures patients’ self-reported experiences rather than actual skills [32], which may overestimate true comprehension and is vulnerable to response biases where participants may wish to appear competent or positive [25]. Finally, the study was conducted within the Norwegian surgical context, with a strong public healthcare infrastructure. Consequently, findings may be restrained to similar clinical healthcare settings.

## Conclusion

The overall findings showed no significant difference in health literacy domain scores following the intervention. The presence of ceiling effects in this relatively high-functioning Norwegian patient population may have masked a true effect, suggesting that the HLQ instrument may be less suitable for evaluating this specific intervention. Nevertheless, an interaction effect with time for the domain “Actively managing my health”, may indicate that the patient-driven checklist could support elective surgical patients’ health management. This finding aligns with qualitative feedback and warrants further investigation using larger samples and more sensitive and context-specific measurement tools.

## Supporting information

S1 File CONSORT 2025 checklist of information to include when reporting a randomised trial. (DOCX)

S2 File Study protocol. (DOCX)

S1 Table Frequencies of the Health Literacy Questionnaire items for domains 1–5. (DOCX)

S2 Table Frequencies of the Health Literacy Questionnaire items for domains 6–9. (DOCX)

S3 Table Sensitivity analysis included mixed-effect model analysis with missing imputation for non-responders (n = 355) for PASC’s impact on the Health Literacy Questionnaire domains. (DOCX)

S4 Table Cronbach’s alpha values and 95% Confidence Intervals for the Health Literacy Questionnaire domains. (DOCX)
