# Supplementary material for: Effect of a patient-driven perioperative intervention on health literacy: A stepped-wedge cluster randomised sub-study
Source: PLoS One. 2026 Jun 24;21(6):e0352245. doi: 10.1371/journal.pone.0352245 (PMC13293430; doi:10.1371/journal.pone.0352245)
Supplement: S2 File — (DOCX) [file pone.0352245.s002.docx]

**S2 File. This is the S2 File Study Protocol**

# 1. Project title

Impact of surgical patients’ safety checklist (PASC) on patients’ health literacy and empowerment

# 2. Introduction

Patient information and health literacy are important factors in achieving optimal surgical outcome and patient safety.^1 2^ However patients are inundated with complex health information for which they receive little guidance on how to interpret.^3^ Personal health literacy is the degree to which individuals have the ability to find, understand, and use information and services to inform health-related decisions and actions for themselves and others, whereas “organizational health literacy” refers to the degree to which organizations equitably enable individuals to find, understand, and use information and services to inform health-related decisions and actions for themselves and others.^4^ Although personal health literacy is considered contextual, health organizations as producers of health information and services, are responsible in improving and equitably address health literacy.^4^

Several studies suggest that enhanced health literacy and patient empowerment might reduce postoperative complications and improve patient’s outcome after surgery.^5-9^ Despite this a recent review reveals that large parts of the surgical patients have low health literacy and that few interventions exist addressing either personal or organizational health literacy development or implementation in surgical care.^10^

Low health literacy in patients might lead to inadequate comprehension of the surgical procedure and discharge instructions.^2^ Poor uptake and understanding of critical healthcare information might thereby represent a major safety risk for surgical patients. In this matter a research review have found several impacts of limited health literacy by surgical patients including longer hospital stay, complications and less adherence to preoperative instructions.^3^ Moreover, low health literacy has been reported to be associated with poorer health, decreased follow-up of one`s own disease and increased mortality.^6 11 12^

Both in developing and developed countries health literacy is highlighted as a key determinant of a person´s ability to optimally manage their health and of a health system´s ability to ensure equitable access to and use of services.^12^ Improved health literacy is a key dimension of Health 2020, the European health policy framework.^13^ The Norwegian government aims to increase health literacy and the Ministry of Health and Care Services will facilitate the increase of health literacy of the population through a number of measures.^12^

Patient empowerment is defined by the World Health Organization as “a process through which people gain greater control over decisions actions affecting their health”.^14^ Further it is claimed that patient empowerment is a complex and participatory process, which aims at improving the health-related knowledge, skills, attitudes and expertise of patients to raise their awareness of their co-creating delivery of care.^15^ Patient empowerment is advocated by several studies to improve healthcare outcomes as well as increase postoperative health-related quality of life,^16-18^ which this project supports. Health literacy and patient empowerment are distinct concepts but closely interwoven and should be considered in conjunction.^16^ Health literacy activates a self-nourishing cycle of patient empowerment, and patients who have good health literacy, are more likely to effectively utilize this knowledge which can potentially empower them to actively participate in their own health care consulations.^19^ Further it is stated that health literacy is included as one indicator of patient empowerment because patients need to understand the information to use it effectively.^20^ Enhanced health literacy can empower patients to participate in own care and safety,^12 21^ hence both concepts will be included in this project.

The World Health Organization states that “only an informed and empowered consumer can actively contribute to improve communication as well as health care outcomes”.^22^ The informed patients also need counterparts, and healthcare professionals should therefore contribute to empower patients by creating the right environment enabling patients to actively participate in their healthcare.^6 23^ This can be accomplished in several ways, for example by welcoming questions, and by encouraging shared decision making and participation in discussions and safety checks.^24 25^ Equally important is the practical need to show patients how they can help to optimise the safety of their care and to provide them with tools and information they require for doing so.^6 26^ The patients´ surgical safety checklist (PASC) has recently been designed with the goal of helping surgical patients to be more aware of what actions they can do to prevent complications and acquire which information they need throughout the surgical pathway.^27^ The themes of the checklist items are: pre-operative information, pre-operative preparations, post-operative information, post-operative plans and follow-up. PASC is designed in two sections, one to be used before surgery and the second prior to discharge from the hospital and can be used as a tool for surgical patients to prepare for surgery and discharge to home and might contribute to empower surgical patients to become more involved in their own safety.^28^

The established PASC project investigate the implementation processes and effect of PASC on complications, mortality, hospitalisation time, and health economics. It is administered by the Department of Anesthesia and Intensive care at Haukeland University Hospital (HUH) and are financed by the Research Council of Norway (ID: 320475). Preliminary results in the PASC project highlight key risk areas as perceived by patients and healthcare workers.^27^ This PhD project will be an additional work package for the PASC research project as a part of a larger stepped wedge cluster randomized controlled trial (SWCRCT) (ClinicalTrials.gov, ID: NCT03105713).

There are several examples of patient-completed safety checklists designed to empower patients to be involved in their own care in surgery.^5 27 29^ However, both quantitative and qualitative studies are needed to more clearly understand the relationship between health literacy and surgical outcomes.^10^ In addition more studies to establish the impact of poor health literacy on perioperative outcomes are needed.^2^ A recent review suggests a need for the awareness of surgical patients’ health literacy in order to promote universal measure of support to ensure adequate communication, patient engagement, and quality of care.^3^ The core objectives of patients’ checklists are to improve patient empowerment to enhance safety,^26^ which this project will investigate. PASC is a new intervention, developed for empowering surgical patients to be involved in their own safety.^27 28^ The PASC trial is currently ongoing in two of the Western Regional Norway Health Authority hospitals, including general-, orthopaedic-, neuro-, cardio-thoracic-, breast- and endocrinology-, ear-nose-throat-, and gastro-intestinal surgical patients (n= 5320). Patients are provided with an electronically checklist (ePASC) and a paper version of it in the trial. In the PASC project, we investigate the impact of the checklist on patient outcomes in three work packages: undernutrition, implementation and economic evaluations.^30^ Beyond these work packages, there is a need for research to add novel insight of the PASC impact on patients’ health literacy and empowerment. The current research gap will be addressed in this PhD project by applying both qualitative and quantitative studies of elective surgical patients´ health literacy levels as well as patients’ empowerment experiences in their surgical pathways, in baseline and intervention groups of the PASC trial.

This PhD project may provide valuable understanding of the impact of PASC on health literacy and empowerment which is shown to enhance the quality and safety of care.^15 31^ Focus group interviews and a validated health literacy questionnaire will be important indicators of the checklist impacts of health literacy and patient empowerment.^32^

## 2.1. Impact on patient care

Health literacy and patient empowerment are recognized as core values of high-quality patient-centred care.^13 33^ By exploring PASC’s impact on surgical patients` experiences of health literacy and empowerment, this project provide novel knowledge and guidance on how patients can be more actively involved in their own surgical care. The advantages of a possible increased health literacy among surgical patients as result of PASC, may include shortening of hospital stays, less complications and increased satisfaction and well-being of the patients as well as increased interaction with the healthcare team.^10^ These benefits may in addition lead to both cost and efficiency benefits for the organization with less complications and shorter hospital stay, less readmissions as well as decreased deletion of operations due to less adherence to preoperative instructions.^3^

The surgeons and other surgical staff may benefit from enhanced health literacy as their patients need to comprehend the nature, risks and benefits of the surgical procedure, adhere to strict perioperative instructions, and make complex care-decisions about interventions.^3^ Surgeons are already involved in the PASC project and will continue to contribute to this project.

Enhanced communication and cooperation between the surgical patients, their surgeon and general practitioner (GP) both before and after surgery might also be an important impact of this project to increase patients´ health outcome and quality of care. To facilitate this, GP representatives from Haukeland University Hospital will participate in this PhD project and in the research of patients´ experiences of health literacy and empowerment.

Other personnel groups in surgical care may also benefit from this project as patients with high health literate are associated with higher satisfaction and well-being.^10^ Because of the current research gap of measured health literacy and patient empowerment by surgical patients this project will bring novel knowledge of this field into the research community. The results of this project may benefit and enhance health literacy and empowerment of future surgical patients. Additional, other patient groups can adapt the checklist concept and adapt it to other parts of healthcare services, for example in medical patients.

One of the United Nations (UN) Sustainable development goal (SDG#3) is to ensure healthy lives and promote well-being for all at all ages. If successful, the PASC will give a significant contribution, reducing patient harm. The project outputs may provide important insight on patient involvement in patient safety and on patients’ health literacy and empowerment to participate.

# 3. Objectives and Goals/Milestones of the Project/Research Questions

The overall aim of this project is to gain knowledge on how the implementation of PASC impacts on health literacy and patient empowerment. This project hypothesizes that PASC implementation enhances elective surgical patients’ health literacy and empowerment, contributing to improved patient involvement and healthcare outcome. To demonstrate possible relationships between the PASC and health literacy, and to explore the patients` experiences, this project will use a combination of quantitative and qualitative research methods. The following research questions will be used:

Research question 1:

- How do elective surgical patients perceive and experience health literacy and empowerment throughout standard surgical care?

Research question 2:

- How do elective surgical patients perceive and experience health literacy and empowerment when having utilized the patients’ safety checklist?

Research question 3:

- Does implementation of the patients’ safety checklist have impact on elective surgical patients’ health literacy? A stepped wedge cluster randomized controlled trial.

Short -term goals: First, to establish knowledge of surgical patients’ experiences regarding health literacy and patient empowerment in baseline and intervention groups of the PASC trial. Second, to identify PASC impact on health literacy measured by the Health Literacy Questionnaire (HLQ).^32^

Long-term goals: First, increase surgical patients’ involvement to improve work processes, quality, and safety in surgical pathways. Second, facilitate implementation of PASC to other surgical wards, specialities, and hospitals to enhance patient’s health literacy, empowerment, and outcomes.

Expected result of this project is to develop increased knowledge of surgical patients’ health literacy needs, which can contribute to enhanced patient empowerment throughout surgical pathways. This knowledge will give us valuable guidance of the impact of PASC on the quality and safety of care. In addition, the results of this project might lead to that other health care specialities are willing to adopt the patient-completed checklist concept to improve patient involvement in health care and safety. Ultimately, this PhD project will contribute increase research competence within the field of advanced nursing specialities as anaesthesia, operating theatre or intensive care nursing, with a candidate within one of these specialties.

Dissemination and this PhD course plan:

| **Timeframe** | **Activities in conjunction with the PASC project timeline** | **Publications** |
| --- | --- | --- |
| June 2022-January 2023 | Data sampling study 1 (seven focus group interviews). Data analysis, writing article 1  Datasampling study 3 (HLQ questionnaire), quality ensure data  PhD program at OsloMet University, Oslo (study points) | Present article 1 abstract at international scientific conferences  Submit article 1 in XXXXXX (level 2) |
| October 2022 – October 2023 | Data sampling study 2 (seven focus group interviews). Data analysis, writing article 2  Data sampling study 3 (HLQ questionnaire), quality ensure data  PhD program at OsloMet University, Oslo (study points) | Present article 2 abstract at international scientific conferences  Submit article 2 in XXXXX (level2) |
| January 2024- December 2024 | Data sampling study 3 (HLQ questionnaire), quality ensure data, data analysis  Writing article 3, complete PhD thesis | Present article 3 abstract  Submit article 3 in XXXXX (level2), PhD thesis |

*Comment: Time frame has been exceeded. Tentative submission of articles to scientific journal was anonymized 12.12.2025, by project leader, Professor Arvid Steinar Haugen.*

# 4.Feasibility

The basic research structures in this project is based on the ongoing PASC project. The established research group ensure the quality and supervision of the study. This PhD project include one focus group interview in each cluster in the PASC trial, both in the control and intervention arms, in total up to 14 interviews. The research group has performed similar interviews in the development and validation phases of PASC, and in our experience, this should be feasible to perform in collaboration with the wards. Similarly, the data collection to evaluate health literacy through the HLQ is is feasible. The numbers of patients responding on the survey (n=350 in baseline/intervention) are based on a power analysis which indicates it is possible to collect data within the study time frame. Hence, implementation of all tasks in this PhD project is feasible within the project period.

## 4.1. Study design, Choice of Methodology and Analysis

The PhD project include three studies which investigate PASC´s impact on health literacy and patient empowerment. The methods used in the two first parts will be qualitative and in the third part quantitative.

- Study 1 and 2. Qualitative studies with one focus group interview of elective surgical patients’ experiences of health literacy and empowerment in each of the seven surgical clusters in the SWCRT baseline phase and similar in the intervention phase.

Study 1 and 2 include focus group interviews of elective surgical patients that are recruited from two Norwegian hospitals, Haukeland University Hospital and Førde community hospital. The focus groups will take place at pre booked suitable premises at these two hospitals. Phone interviews are an option depending on the pandemic situation. The inclusion criteria are both genders, age 18 or above, fluent in Norwegian, cognitive capable to participate in a focus group interview, living at home (no institution), and living within approximately one hour drive from the hospital.

There will be 7 focus group interviews in study 1 and 7 interviews in study 2. Each interview will include 6-8 participants. Focus group interviews are considered as an appropriate way to investigate patient’s experiences using a specific intervention.^34^ Focus groups can give a deeper understanding of the surgical patients’ perceptions and experiences of health literacy and empowerment by encouraging a discussion around the issues between the patients.^34^ Focus groups have the advantage of enabling a researcher to obtain the viewpoint of a larger group in a short time.^34^ The analyses will have an inductive approach, and content analysis according to Graneheim & Lundeman´s method will be used.^35^ This includes description of the manifest content, close to the text, as well as interpretations of the latent content, distant from the text but still close to the participants lived experiences.^36^ During this inductive approach, researchers will search for patterns, and describe similarities and differences in the data in categories and themes. Trusthworthiness, credibility, dependency, and transferability of the results will in line with Graneheim & Lundeman,^34^ be discussed in the study 1 and 2.

- Study 3. This study investigate if there are any differences between patients’ health literacy scores in the baseline cluster steps and patients’ in the intervention steps of the trial. The validated Health Literacy Questionnaire (HLQ),^37^ is the primary outcome for elective surgical patients´ health literacy scores.

A Stepped Wedge Cluster RCT design is considered as a robust research design when investigate patient safety interventions.^38 39^ The inclusion criteria’s of study 3 equals the ones of study 1 and 2, except for living within one hour from the hospital. The HLQ which is translated and validated in a Norwegian population.^32^ A license has been provided for using HLQ in this project. The HLQ consists of 44 questions divided in to 9 domains designed to measure the patients’ health literacy. 1: Understood and supported by health professionals. 2: Satisfactory information to handle own health. 3: Active management of own health. 4: Social support of own health. 5: Consider health information. 6: Active cooperation with health professionals. 7: Navigation through the healthcare system. 8: Possibility’s to find right health information and 9: Understand the health information to be able to know what should be done.^32^

The required number of participants in study 3 was calculated in a power analysis performed by Professor in biostatistics, Roy Miodini Nilsen. The lowest number of individuals required to detect a clinical meaningful mean score difference at 0.30 between the two groups was n = 103 patients in each arm of the PASC trial. Based on the validation study of HLQ standard deviation at 0.66 in both arms were assumed.^32^ Power was estimated at 90% and type I error at 0.05. It was estimated from previous research experiences from the same surgical samples that the HLQ response could possibly be as low as 30%, therefore n is set at 350 for each group. The research group/PhD candidate will thereby send prepaid envelopes with a query of signing the consent form, and answering the HLQ to elective surgical patients, 50 x 7 clusters in both arms of the trial.

Descriptive statistics will be used to describe patient characteristics. A chi-squared test will be used to test for differences between the trial arms. Any differences on patient characteristics will be adjusted for by using linear regression comparing effects across the nine domains on health literacy in the control and intervention arms.^40^ All analysis will be performed in SPSS 26.0 (IBM Corp, Armonk, NY) and a P value at 0.05 will be considered statistical significant, with a two-tailed test.

The PASC research group and the PhD candidate is based within the infrastructures at the Department of Anaesthesia and Intensive Care, at Haukeland University Hospital. The candidate will have access to all equipment and databases needed to carry out this research project. The PhD candidate will be part of the established PASC research group, led by Professor Arvid Steinar Haugen. The candidate have to meet the criteria for uptake at the PhD program at OsloMet, Faculty of Health Sciences, Department of Nursing and Health Promotion, Acute and Critical Illness.

A key challenge of the project might be the pandemic hindering execution of the focus groups, although this risk has been managed successfully with adequate precautions. This was solved with booking of big enough meeting room to achieve satisfactory distance between the participants with hand disinfection easily accessible. Other solutions for example online interviews might also be necessary if the physical meetings are not an option due to the pandemic. The response rate to the questionnaires may be a challenge for the study. However, with the resources available within the PASC project and with the hospitals’ collaboration, recruitment of the desired number of patients is feasible to achieve.

The PhD fellow will be responsible for:

- Recruiting participants in all three studies together with the rest of the PASC research team.
- Carrying out the focus groups interviews and analysing the data in cooperation with supervisors and the PASC research team.
- Sending the questionnaires to the participants and ensuring the collection, registration, and integrity of the data.
- Publishing as a first author of three articles. The two first articles aims to be published in level 2 journals as i.e. BMC Health Services Research and BMJ Quality & Safety.
- Presentation of the results in three international scientific conferences.

## 4.2. Organisation and Collaboration

The PhD project will be part of a larger research project PASC, which is funded by the Norwegian Research Council (ID 320475). The administrator of the PASC project is clinical director Hanne Klausen and led by Professor Arvid Steinar Haugen. PASC is based at the Department of Anaesthesia and Intensive Care, at Haukeland University Hospital (HUH). The research group has a long record of studies on surgical safety checklists. Department of Surgery, Helse Førde, general practitioners Dr Bhaumik Thakkar and Dr Kjetil Hagerup, Helse Vest IKT and CheckWare, and the Norwegian Institute of Public Health are PASC project partners. The project collaborates with seven included surgical departments in Helse Bergen and Helse Førde and are supported by the competence centre for clinical research at the department of research and innovation, Helse Bergen. The PASC project also collaborate with Kings College (London), department for clinical nutrition, HUH, Helse Førde, Helse Vest IKT, University in Bergen, Norwegian Institute of Public Health, and is endorsed by UKOM. The candidate will be supervised by:

Arvid Steinar Haugen, Professor at OsloMet, is the principal investigator (PI) of PASC and will be the PhD candidate’s main supervisor. Haugen is Head Nurse at the Department of Anesthesia and Intensive Care at HUH. He has advanced research expertise in patient safety, safety culture and specialized expertise in implementation of safety checklists, including SWCRCTs (H-index ResearchGate: 12). He has 25 peer-reviewed publications, eight book chapters, supervises 6 PhD candidates (3 defended) and 26 Master students.

Co-supervisor of the PhD candidate will be Eirik Søfteland, Professor (UIB) and senior consultant in anesthesiology (HUH). He has 38 peer-reviewed publications and has supervised 6 PhD candidates (H-index ResearchGate: 15). At present Søfteland leads the Norwegian Research Council funded IMPLEMENT-IT study and together with Professor Stig Harthug the established Bergen Surgical Checklist study group. The PASC and IMPLEMENT-IT will form a collaborative research group. Hilde Valen Wæhle, PhD, participatory PASC researcher (post-doctor level) will also be co-supervisor for the PhD candidate. She is quality advisor at the Department of Research and Development at HUH and holds a research position at Centre for Resilience in Healthcare (SHARE), University of Stavanger. She has 6 peer-reviewed publications on patient safety and surgical safety checklist.

Kristin Harris, PhD candidate at UIB, has developed and validated the PASC checklist in her PhD project. She is part of the supervising team and the PASC research group that will support the new PhD candidate.

The PhD candidate will be a part of the PASC research group and participate in all research planning meetings regarding the project. Further, the candidate participates in the Network for Patients Safety Research supported by the Western Norway Regional Health Authority trust.

Overview of PhD project organization:

| Name, title, affiliation | Expertise | Role | Contribution to studies |
| --- | --- | --- | --- |
| XXXXXXXXX | Master in XXXXXXXXXXXXX | PhD candidate | Study 1-3 |
| Arvid Haugen, Professor, HUH and OsloMet | Project leader and Principal Investigator of PASC | Main supervisor | Study 1-3 |
| Hilde Valen Wæhle, PhD, HUH | PASC researcher on implementation processes, patient experiences, and qualitative methodology | Co-supervisor | Study 1-3 |
| Kristin Harris, MCCN, HUH | Development and validation of PASC/PhD project | Project partner | Study 1-3 |
| Eirik Søfteland, Professor, HUH, UIB | Leader of the Bergen Surgical Checklist Study and Implementation research group | Co-supervisor | Study 1-3 |
| Anette Storesund, PhD, HUH | PASC researcher on patient outcomes | Project partner | Study 1-3 |
| Roy Miodini Nilsen, Professor | Biostatistician | Statistical advisor | Study 3 |
| Nick Sevdalis, Dept. of Anaesthesia and Intensive care, HUH; Professor and Director of Centre of Implementation Science, Kings College London, UK | International expert in implementation sciences and research on patient safety in healthcare | International project partner | Study 1-3 |
| Bhaumik Thakkar, GP, Morvik Legesenter | Representative of GPs at Haukeland University Hospital | Project partner | Study 2 |
| Torhild Næss Vedeler, HUH  John-Helge Heimdal, HUH  Evelyn Neppelberg, HUH  Kjell Vikenes, HUH  Tom Guldhav, Helse Førde  Hanne Klausen, HUH | Clinical director, Neuro clinic  Clinical director, Surgical clinic  Clinical director, Head and Neck clinic  Clinical director, Cardio-thoracic clinic  Director, Surgical clinic  Clinical director, Anaesthesia and Intensive Care | Project partners  Project administrator | Study 1-3 |
| User representatives HUH | User experiences in surgery | Project participants | Study 1-3 |

4.3 PhD Plan and Milestones
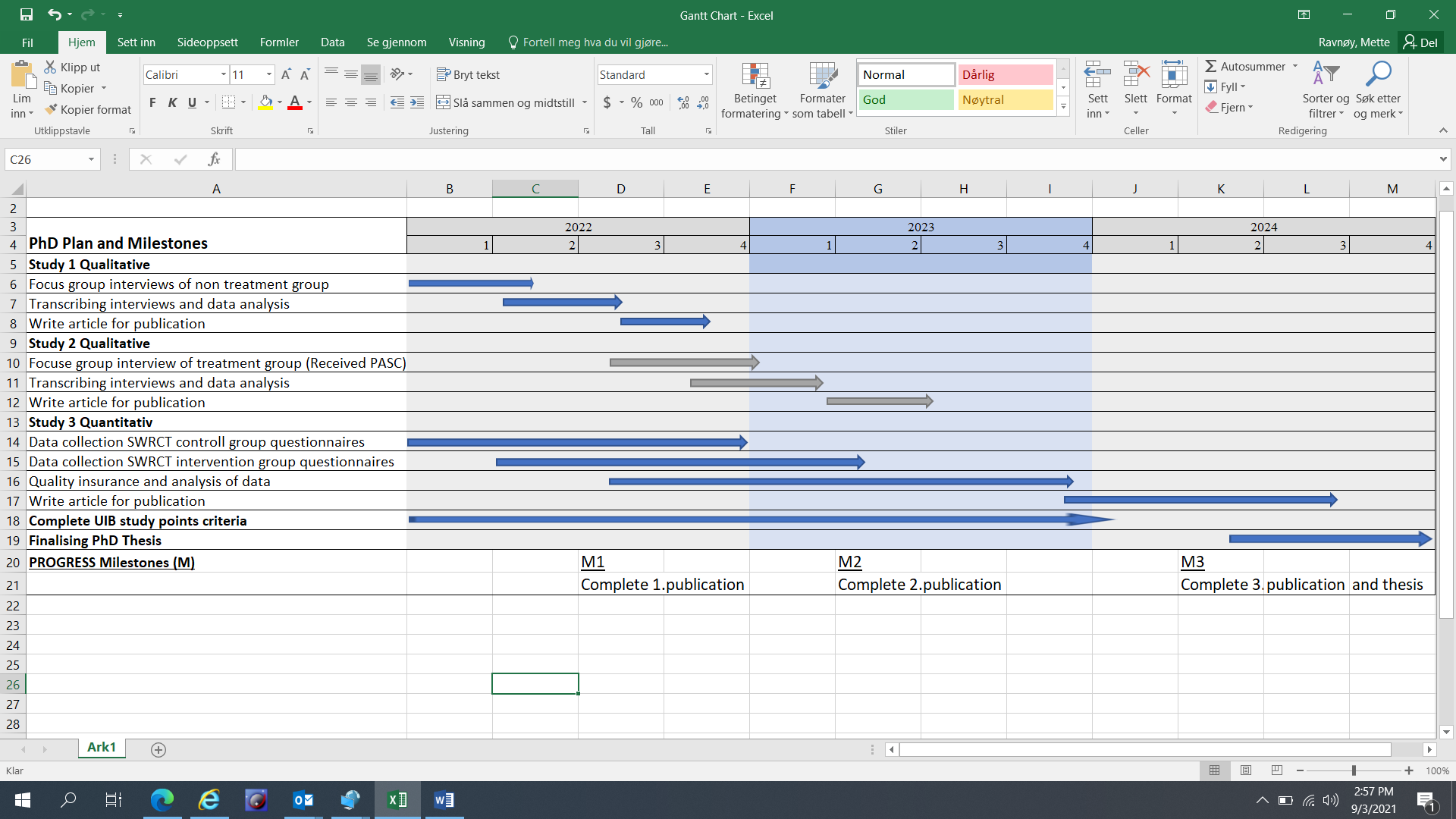


## The result of this project will be structured for publication of three scientific articles, primarily at level 2. The PhD candidate will be the first author of all papers.

- Article 1. Surgical patients’ experience of health literacy and empowerment.
- Article 2. Surgical patients’ health literacy and empowerment after using PASC – a qualitative study.
- Article 3. PASCs impact on health literacy – a stepped wedge cluster RCT.

In addition, oral presentation and posters will be presented at least at three international scientific conferences.

## 4.5. Plans for implementation

The project results will be both published and presented in the research community to facilitate enhanced health literacy and empowerment in surgical patients. Health care personnel involved in surgical patients can use the results to meet the patients’ needs to improve their health literacy and thereby empower the patients to take an active role for their own safety. The result of this project might also facilitate enhanced communication and cooperation between GP, patients, and the surgical wards before and after operation which can increase patients’ preparations and follow-up after surgery. Thereby a GP will participate in the research process including exploration of patients’ experiences of health literacy and empowerment before and after surgery.

Since there are a current gap in health literacy and empowerment evaluation of patient centred safety checklist, this project got an innovation potential of revealing new valuable knowledge to this field. Enhanced knowledge regarding patients’ experiences and levels of health literacy and empowerment by using PASC can also be adopted to other health related fields. The project will contribute to build research competence within the advanced nursing specialities and the care serviced provided by our professional groups, in line with this call.

This PhD research project includes measurement of health literacy levels in surgical patients as well as exploration of the patients’ experiences of both health literacy and empowerment regarding the use of PASC. Future research should focus on health literacy in other surgical populations and how patient empowerment can have impact on patients´ safety in healthcare. The result of this PhD project can support and strengthen future research were PASC implementation can be up scaled to national and international effects studies across not only the surgical field, but also other health care fields.

Plan for implementation-Dissemination to stakeholders:

| **Steakholder** | **What content?** | **In what form?** |
| --- | --- | --- |
| Research Community | Research results, methodology | Three publications, 1. & 2. in BMC Health Services Research, and 3. in BMJ Quality & Safety. Presentations at three international conferences |
| Clinicians | Research results, new recommendations | Presentation at national conferences |
| Politicians | Research results, recommendations | A report to the Directorate of Health, Patient Safety Unit |
| General public | Awareness of the research results | Articles in newspapers, social media posts. |
| Patients | Research results, recommendations | Articles on patient organisation website and social media |

# 5. User Involvement

The very first suggestion to develop the PASC came from the patient safety Ombudsman in Hordaland, Rune Skjælaaen, during an interview in a preceding checklist project, in 2012. User involvement has been highly prioritized throughout the planning, development, and validation

of PASC and this commitment continues in this PhD project. The user representatives at HUH are a part of the PASC research development process and have been involved in meetings and giving feedback throughout the project development. The users’ experiences are vital both to help researcher identifying health literacy and empowerment experiences by surgical patients and a useful resource to develop the best interview guide suitable for this study.

The user perspective, needs and experiences provide essential information for the research, and their involvement is a priority in this project. The user representatives of HUH has piloted and shared their insight on the checklist and provided input to the interview guide prior to focus group interviews of patients. They will further be challenged to participate in implementation of the research result.

# 6. Ethical Consideration

The study will follow the Helsinki declaration’s research principles.^41^ The Regional Committee for Medical and Health Research Ethics (REC West) of the Western Norway Health Region (2016/1102) have approved the PASC study prior to study start. The project is approved by the hospital administration within the two hospitals involved.

Patients in the study will be given verbal and written information about the study at recruitment. The participants will be informed that their participation in the study is voluntary and that they could withdraw at any time without consequences. All participants will sign an informed consent form. Data will be made anonymous and secured in the research database of HUH with data access only to the primary investigators. The PASC project is registered in Clinical Trials.gov: NCT03105713.

# 7. References

1. World Health Organization. Patient information for surgical safety: what you need to know before and after surgery. Genova: World Health Organization, 2015.

2. De Oliveira JGS, McCarthy RJ, Wolf MS, et al. The impact of health literacy in the care of surgical patients: a qualitative systematic review. *BMC Surg* 2015;15(1):86-86. doi: 10.1186/s12893-015-0073-6

3. Roy M, Corkum JP, Urbach DR, et al. Health Literacy Among Surgical Patients: A Systematic Review and Meta-analysis. *World J Surg* 2019;43(1):96-106. doi: 10.1007/s00268-018-4754-z

4. Hussey M, Hughes J. Design and analysis of stepped wedge cluster randomized trials. *Contemparary Clinical Trials* 2006

5. Russ S, Latif Z, Hazell AL, et al. A Smartphone App Designed to Empower Patients to Contribute Toward Safer Surgical Care: Community-Based Evaluation Using a Participatory Approach. *JMIR Mhealth Uhealth* 2020;8(1):e12859-e59. doi: 10.2196/12859

6. Kickbusch I, Pelikan JM, Apfel FA, Tsouros D. World Health Organization. Regional Office for Europe. Health literacy: the solid facts. Copenhagen. 2013:73. [accessed 03.13 2022].

7. Mitchell SE, Sadikova E, Jack BW, et al. Health Literacy and 30-Day Postdischarge Hospital Utilization. *J Health Commun* 2012;17(sup3):325-38. doi: 10.1080/10810730.2012.715233

8. Collette A-M, Spies C, Eckardt R, et al. Patient Empowerment Reduces Pain in Geriatric Patients After Gynecologic Onco-Surgery: Subgroup Analysis of a Prospective Randomized Controlled Clinical Trial. *J PeriAnesth Nurs* 2018;33(3):281-89. doi: https://doi.org/10.1016/j.jopan.2016.10.005

9. Schmidt M, Eckardt R, Scholtz K, et al. Patient Empowerment Improved Perioperative Quality of Care in Cancer Patients Aged ≥ 65 Years - A Randomized Controlled Trial. *PLoS One* 2015;10(9):e0137824-e24. doi: 10.1371/journal.pone.0137824

10. Chang ME, Baker SJ, Dos Santos Marques IC, et al. Health Literacy in Surgery. *Health Lit Res Pract* 2020;4(1):e46-e65. doi: 10.3928/24748307-20191121-01

11. Osborne RH, Batterham RW, Elsworth GR, et al. The grounded psychometric development and initial validation of the Health Literacy Questionnaire (HLQ). *BMC Public Health* 2013;13(1):658. doi: 10.1186/1471-2458-13-658

12. Helse- og omsorgsdepartementet. Strategi for å øke helsekompetansen i befolkningen 2019-2023, 2019.

13. World Health Organization. Health 2020: a European policy framework and strategy for the 21st century. Copenhagen: World Health Organization. Regional Office for Europe 2013:182.

14. World Health Organization. Health promotion glossary. Geneva: World Health Organization, 1998.

15. Palumbo R. The Bright Side and the Dark Side of Patient Empowerment: Co-creation and Co-destruction of Value in the Healthcare Environment. 1st ed. 2017. ed. Cham: Springer International Publishing : Imprint: Springer, 2017.

16. Schulz PJ, Nakamoto K. Health literacy and patient empowerment in health communication: The importance of separating conjoined twins. *Patient Education and Counseling* 2013;90(1):4-11. doi: https://doi.org/10.1016/j.pec.2012.09.006

17. Koekenbier K, Leino-Kilpi H, Cabrera E, et al. Empowering knowledge and its connection to health-related quality of life: A cross-cultural study: A concise and informative title: Empowering knowledge and its connection to health-related quality of life. *Appl Nurs Res* 2016;29:211-16. doi: 10.1016/j.apnr.2015.05.004

18. Jerofke T, Weiss M, Yakusheva O, et al. Patient perceptions of patient-empowering nurse behaviours, patient activation and functional health status in postsurgical patients with life-threatening long-term illnesses. *J Adv Nurs* 2014;70(6):1310-22. doi: 10.1111/jan.12286

19. Edwards M, Davies M, Edwards A, et al. What are the external influences on information exchange and shared decision-making in healthcare consultations: A meta-synthesis of the literature. *Patient Educ Couns* 2008;75(1):37-52. doi: 10.1016/j.pec.2008.09.025

20. Londoño AMM, Schulz PJ, Londoño AMM, et al. Influences of health literacy, judgment skills, and empowerment on asthma self-management practices. *Patient Educ Couns* 2015;98(7):908-17. doi: 10.1016/j.pec.2015.03.003

21. World Health Organization. Regional Office for South-East A. Health literacy toolkit for low- and middle-income countries: A series of information sheets to empower communities and strengthen health systems. New Delhi: WHO Regional Office for South-East Asia 2015.

22. World Health Organization. Exploring patient participation in reducing health-care-related safety risks WHO Regional Office for Europe2013 [cited 2021 20.01]. Available from: [http://www.euro.who.int/__data/assets/pdf_file/0010/185779/e96814.pdf accessed 20.01 2021](http://www.euro.who.int/__data/assets/pdf_file/0010/185779/e96814.pdf%20accessed%2020.01%202021).

23. Bonsignore C, Brolis E, Lonescu A, et al. Patient empowerment and centredness: European Health Parliament;

[EHP-papers_Patients-empowerment.pdf (healthparliament.eu)](https://www.healthparliament.eu/wp-content/uploads/2017/09/EHP-papers_Patients-empowerment.pdf) [accessed 12.03. 2022]

24. Davis RE, Sevdalis N, Pinto A, et al. Patients’ attitudes towards patient involvement in safety interventions: results of two exploratory studies. *Health Expectations* 2013;16(4):e164-e76. doi: 10.1111/j.1369-7625.2011.00725.x

25. Trier H, Valderas JM, Wensing M, et al. Involving patients in patient safety programmes: A scoping review and consensus procedure by the LINNEAUS collaboration on patient safety in primary care. *European Journal of General Practice* 2015;21:56-61. doi: 10.3109/13814788.2015.1043729

26. Harris KR, Russ S. Patient-completed safety checklists as an empowerment tool for patient involvement in patient safety: concepts, considerations and recommendations. *Future Healthcare Journal* 2021;8(3): e567–73.

27. Harris K, Søfteland E, Moi AL, et al. Patients’ and healthcare workers’ recommendations for a surgical patient safety checklist – a qualitative study. *BMC Health Services Research* 2020;20(1):43. doi: 10.1186/s12913-020-4888-1

28. Harris K, Søfteland E, Moi AL, et al. Development and validation of patients’ surgical.safety checklist. *BMC Health Serv* *Res* 2022. 22: 259. doi: 10.1186/s12913-022-07470-z

29. Hardiman KM, Reames CD, McLeod MC, et al. Patient autonomy–centered self-care checklist reduces hospital readmissions after ileostomy creation. *Surgery* 2016;160(5):1302-08. doi:https://doi.org/10.1016/j.surg.2016.05.007

30. Research Council of Norway. Implementation of Patients Safety Checklist (PASC) in Surgery, a Stepped Wedge Cluster RCT - Effects on Patient and Implementation Outcomes Project Bank: Research Council of Norway; 2021 [Available from: <https://prosjektbanken.forskningsradet.no/en/project/FORISS/320475?Kilde=FORISS&distribution=Ar&chart=bar&calcType=funding&Sprak=no&sortBy=date&sortOrder=desc&resultCount=30&offset=0&Organisasjon.3=HUSEBY+G%C3%85RD+Amund+Huseby>] [accessed 03.12.2022 2022}.

31. Kickbusch I, Pelikan JM, Apfel FA, Tsouros D. World Health Organization. Regional Office for Europe. Health literacy: the solid facts. Copenhagen. 2013:73.

32. Wahl AK, Hermansen Å, Osborne RH, et al. A validation study of the Norwegian version of the Health Literacy Questionnaire: A robust nine-dimension factor model. *Scand J Public Health* 2020;49(4):471-78. doi: 10.1177/1403494820926428

33. Pekonen A, Eloranta S, Stolt M, et al. Measuring patient empowerment - A systematic review. *Patient Educ Couns* 2020;103(4):777-87. doi: 10.1016/j.pec.2019.10.019 [

34. Polit DF, Beck CT. Nursing Research : generating and assessing evidence for nursing practice. 10th ed. ed. Philadelphia: Wolters Kluwer 2017.

35. Graneheim UH, Lundman B. Qualitative content analysis in nursing research: concepts, procedures and measures to achieve trustworthiness. *Nurse Educ Today* 2004;24(2):105-12. doi: 10.1016/j.nedt.2003.10.001

36. Graneheim UH, Lindgren B-M, Lundman B. Methodological challenges in qualitative content analysis: A discussion paper. *Nurse Educ Today* 2017;56:29-34. doi: 10.1016/j.nedt.2017.06.002

37. Osborne RH, Batterham RW, Elsworth GR, et al. The grounded psychometric development and initial validation of the Health Literacy Questionnaire (HLQ). *BMC Public Health* 2013;13(1):658-58. doi: 10.1186/1471-2458-13-658

38. Torgerson D. Designing randomised trials in health, education and the social sciences: an introduction: Springer 2008.

39. Mdege ND, Man M-S, Taylor CA, et al. Systematic review of stepped wedge cluster randomized trials shows that design is particularly used to evaluate interventions during routine implementation. *J Clin Epidemiol* 2011;64(9):936-48. doi: http://dx.doi.org/10.1016/j.jclinepi.2010.12.003

40. Altman DG. Practical statistics for medical research. London: Chapman & Hall/CRC 2018.

41. World Medical Association. World Medical Association Declaration of Helsinki: Ethical Principles for Medical Research Involving Human Subjects. *JAMA* 2013;310(20):2191-94. doi: 10.1001/jama.2013.281053
