# Supplementary material for: Effect of a patient-driven perioperative intervention on health literacy: A stepped-wedge cluster randomised sub-study
Source: PLoS One. 2026 Jun 24;21(6):e0352245. doi: 10.1371/journal.pone.0352245 (PMC13293430; doi:10.1371/journal.pone.0352245)
Supplement: S1 Table — (DOCX) [file pone.0352245.s003.docx]

| **S1 Table. This is the S1 Table The Frequencies of the Health Literacy Questionnaire Items for Domains 1-5** | | | | | | | | |
| --- | --- | --- | --- | --- | --- | --- | --- | --- |
| Health Literacy Questionnaire Domains (D) and Items | **Control (n=137)** | | | |  | | | |
|  |  |  |  |  | **Intervention (n=208)** | | | |
|  | Strongly disagree | Disagree | Agree | Strongly agree | Strongly disagree | Disagree | Agree | Strongly agree |
|  | n (%) | n (%) | n (%) | n (%) | n (%) | n (%) | n (%) | n (%) |
| **D1.** **Feeling understood and supported by healthcare providers** | | | | | | | | |
| 1. I have at least one healthcare provider who… | 8 (6.1) | 15 (11.4) | 66 (50.0) | 43 (32.6) | 10 (4.9) | 49 (23.8) | 79 (38.3) | 68 (33.0) |
| 2. I have at least one healthcare provider I can… | 5 (3.7) | 12 (8.9) | 84 (62.2) | 34 (25.2) | 7(3.4) | 35 (16.8) | 105 (50.5) | 61 (29.3) |
| 3. I have the healthcare providers I need… | - | 25 (18.7) | 82 (61.2) | 27 (20.1) | 3 (1.4) | 46 (22.1) | 118 (56.7) | 41 (19.7) |
| 4. I can rely on at least one… | - | 8 (5.9) | 90 (66.2) | 38 (27.9) | 5 (2.4) | 18 (8.7) | 117 (56.3) | 68 (32.7) |
| **D2. Having sufficient information to manage my health** | | | | | | | | |
| 1. I feel I have good information about health… | - | 3 (2.3) | 92 (70.2) | 36 (27.5) | - | 7 (3.4) | 135 (65.2) | 65 (31.4) |
| 2. I have enough information to help me deal… | - | 20 (14.8) | 92 (68.1) | 23 (17.0) | 6 (2.9) | 42 (20.5) | 123 (60.0) | 34 (16.6) |
| 3. I am sure I have all the information I… | - | 32 (23.9) | 79 (59.0) | 23 (17.2) | 2 (1.0) | 59 (28.5) | 120 (58.0) | 26 (12.5) |
| 4. I have all the information I need to | - | 31 (23.0) | 83 (61.5) | 21 (15.6) | 2 (1.0) | 52 (25.1) | 119 (57.5) | 34 (16.4) |
| **D3. Actively managing my health** | | | | | | | | |
| 1. I spend quite a lot of time actively managing… | 1 (0.8) | 27 (20.5) | 76 (57.6) | 28 (21.2) | - | 33 (15.9) | 124 (59.6) | 51 (24.5) |
| 2.I make plans for what I need to do to be… | - | 23 (17.2) | 88 (65.7) | 23 (17.2) | 1 (0.5) | 38 (18.3) | 127 (61.1) | 42 (20.2) |
| 3. Despite other things in my life, I make time… | 1 (0.7) | 27 (20.0) | 83 (61.5) | 24 (17.8) | 2 (1.0) | 34 (16.4) | 137 (66.2) | 34 (16.4) |
| 4. I set my own goals about health and fitness | - | 14 (10.4) | 95 (70.4) | 26 (19.3) | - | 23 (11.1) | 150 (72.1) | 35 (16.8) |
| 5. There are things that I do regularly… | 2 (1.5) | 17 (12.6) | 82 (60.7) | 34 (25.2) | - | 22 (10.6) | 133 (64.3) | 52 (25.1) |
| **D4. Social Support for health** | | | | |  | | | |
| 1. I can get access to several people who… | 1 (0.8) | 21 (16.2) | 75 (57.7) | 33 (25.4) | 5 (2.4) | 44 (21.4) | 106 (51.5) | 51 (24.8) |
| 2. When I feel ill, the people around me really… | 1 (0.8) | 24 (18.3) | 90 (68.7) | 16 (12.2) | 12 (5.8) | 49 (23.8) | 121 (58.7) | 24 (11.7) |
| 3. If I need help, I have plenty of people I… | 2 (1.5) | 20 (14.8) | 82 (60.7) | 31 (23.0) | 3 (1.4) | 35 (16.8) | 112 (53.8) | 58 (27.9) |
| 4. I have at least one person… | 1 (0.7) | 22 (16.2) | 69 (50.7) | 44 (32.4) | 8 (3.9) | 18 (8.7) | 109 (52.7) | 72 (34.8) |
| 5. I have strong support from family or friends | - | 8 (5.9) | 81 (59.6) | 47 (34.6) | - | 10 (4.8) | 108 (52.2) | 89 (43.0) |
| **D5. Appraisal of health information** | | | | |  | | | |
| 1. I compare health information from different | 6 (4.6) | 44 (33.8) | 64 (49.2) | 16 (12.3) | 12 (5.8) | 68 (32.9) | 101 (48.8) | 26 (12.6) |
| 2. When I see new information about health, I… | 8 (6.2) | 39 (30.0) | 71 (54.6) | 12 (9.2) | 7 (3.4) | 70 (33.7) | 101 (48.6) | 30 (14.4) |
| 3. I always compare health information from… | 7 (5.2) | 47 (35.1) | 71 (53.0) | 9 (6.7) | 12 (5.8) | 83 (40.1) | 95 (45.9) | 17 (8.2) |
| 4. I know how to find out if the health… | 3 (2.2) | 32 (23.9) | 80 (59.7) | 19 (14.2) | 2 (1.0) | 66 (31.9) | 109 (52.7) | 30 (14.5) |
| 5. I ask healthcare providers about the quality… | 5 (3.8) | 64 (48.1) | 48 (36.1) | 16 (12.0) | 13 (6.3) | 87 (41.8) | 83 (39.9) | 25 (12.0) |
