# Supplementary material for: Effect of a patient-driven perioperative intervention on health literacy: A stepped-wedge cluster randomised sub-study
Source: PLoS One. 2026 Jun 24;21(6):e0352245. doi: 10.1371/journal.pone.0352245 (PMC13293430; doi:10.1371/journal.pone.0352245)
Supplement: S2 Table — (DOCX) [file pone.0352245.s004.docx]

| **S2** **Table. This is the S2 Table Frequencies of the Health Literacy Questionnaire Items for Domains 6-9** | | | | | | | | | | |
| --- | --- | --- | --- | --- | --- | --- | --- | --- | --- | --- |
| Health Literacy Questionnaire domains (D) and items | **Control (n=137)** | | | | |  | | | | |
|  |  |  |  |  |  | **Intervention (n=208)** | | | | |
|  | Cannot do or always difficult | Usually  difficult | Sometimes difficult | Usually easy | Always easy | Cannot do or always difficult | Usually  difficult | Sometimes difficult | Usually easy | Always easy |
|  | n (%) | n (%) | n (%) | n (%) | n (%) | n (%) | n (%) | n (%) | n (%) | n (%) |
| **D6. Ability to actively engage with healthcare providers** | | | | | |  | | | | |
| 1. Make sure that healthcare providers understand… | - | 4 (3.0) | 45 (33.8) | 69 (51.9) | 15 (11.3) | 1 (0.5) | 17 (8.3) | 60 (29.3) | 109 (53.2) | 18 (8.8) |
| 2. Feel able to discuss your health concerns with a… | - | 6 (4.5) | 27 (20.5) | 73 (55.3) | 26 (19.7) | 3 (1.4) | 15 (7.4) | 38 (18.6) | 106 (52.0) | 42 (20.6) |
| 3. Have good discussions about your health… | - | 8 (6.0) | 26 (19.4) | 70 (52.2) | 30 (22.4) | 3 (1.5) | 15 (7.3) | 47 (22.9) | 100 (48.8) | 40 (19.5) |
| 4. Discuss things with healthcare providers… | 1 (0.7) | 3 (2.2) | 33 (24.6) | 84 (62.7) | 13 (9.7) | - | 8 (3.9) | 46 (22.7) | 113 (55.7) | 36 (17.7) |
| 5. Ask healthcare providers questions to get… | - | 6 (4.4) | 38 (27.7) | 73 (53.3) | 20 (14.6) | 2 (1.0) | 9 (4.4) | 59 (28.9) | 104 (51.0) | 30 (14.7) |
| **D7. Navigating the healthcare system** | | | | | |  | | | | |
| 1. Find the right healthcare | 1 (0.7) | 7 (5.2) | 43 (31.9) | 72 (53.3) | 12 (8.9) | 2 (1.0) | 8 (3.9) | 72 (35.1) | 101 (49.3) | 22 (10.7) |
| 2. Get to see the healthcare providers I need to | - | 9 (6.7) | 45 (33.3) | 66 (48.9) | 15 (11.1) | 2 (1.0) | 22 (10.7) | 62 (30.2) | 90 (43.9) | 29 (14.1) |
| 3. Decide which healthcare provider you need… | 1 (0.8) | 13 (9.8) | 45 (34.1) | 63 (47.7) | 10 (7.6) | 4 (2.0) | 15 (7.4) | 77 (37.7) | 93 (45.6) | 15 (7.4) |
| 4. Make sure you find the right place to get… | - | 5 (3.7) | 42 (31.1) | 75 (55.6) | 13 (9.6) | 1 (0.5) | 10 (4.9) | 77 (37.6) | 94 (45.9) | 23 (11.2) |
| 5. Find out what healthcare services you are… | 2 (1.5) | 22(16.2) | 50 (36.8) | 58 (42.6) | 4 (2.9) | 6 (2.9) | 28 (13.7) | 89 (43.6) | 71 (34.8) | 10 (4.9) |
| 6. Work out what is the best care for you | - | 10 (7.5) | 62 (46.3) | 51 (38.1) | 11 (8.2) | 1 (0.5) | 17 (8.3) | 88 (43.1) | 84 (41.2) | 14 (6.9) |
| **D8. Ability to find good health information** | | | | | |  | | | | |
| 1. Find information about health problems | - | 6 (4.5) | 41 (30.6) | 77 (57.5) | 10 (7.5) | - | 17 (8.3) | 73 (35.8) | 96 (47.1) | 18 (8.8) |
| 2. Find health information from several… | 1 (0.7) | 8 (5.9) | 51 (37.8) | 62 (45.9) | 13 (9.6) | 3 (1.5) | 19 (9.3) | 72 (35.1) | 90 (43.9) | 21 (10.2) |
| 3. Get information about health so you are… | - | 3 (2.2) | 47 (35.1) | 70 (52.2) | 14 (10.4) | - | 14 (6.8) | 76 (37.1) | 94 (45.9) | 21 (10.2) |
| 4. Get health information in words you… | - | 10 (7.4) | 58 (42.6) | 58 (42.6) | 10 (7.4) | - | 12 (5.9) | 77 (37.7) | 94 (46.1) | 21 (10.3) |
| 5. Get health information by yourself | 1 (0.7) | 10 (7.4) | 53 (39.3) | 63 (46.7) | 8 (5.9) | 4 (2.0) | 10 (4.9) | 73 (35.8) | 103 (50.5) | 14 (6.9) |
| **D9. Understanding health information well enough to know what to do** | | | | | |  | | | | |
| 1.Confidently fill medical forms in the correct… | 1 (0.7) | 8 (5.9) | 36 (26.7) | 71 (52.6) | 19 (14.1) | - | 10 (4.9) | 66 (32.2) | 89 (43.4) | 40 (19.5) |
| 2. Accurately follow the instructions from… | - | 3 (2.3) | 20 (15.0) | 81 (60.9) | 29 (21.8) | - | 2 (1.0) | 41 (20.0) | 120 (58.5) | 42 (20.5) |
| 3. Read and understand written health… | 2 (1.5) | 11 (8.2) | 32 (23.9) | 67 (50.0) | 22 (16.4) | 1 (0.5) | 11 (5.4) | 58 (28.4) | 103 (50.5) | 31 (15.2) |
| 4. Read and understand all the information on… | - | 12 (8.9) | 48 (35.6) | 59 (43.7) | 16 (11.9) | - | 10 (4.9) | 66 (32.4) | 95 (46.6) | 33 (16.2) |
| 5. Understand what healthcare providers are… | - | 2 (1.5) | 21 (15.3) | 95 (69.3) | 19 (13.9) | 1 (0.5) | 1 (0.5) | 34 (16.7) | 134 (65.7) | 34 (16.7) |
