# Supplementary material for: Effect of a patient-driven perioperative intervention on health literacy: A stepped-wedge cluster randomised sub-study
Source: PLoS One. 2026 Jun 24;21(6):e0352245. doi: 10.1371/journal.pone.0352245 (PMC13293430; doi:10.1371/journal.pone.0352245)
Supplement: S3 Table — (DOCX) [file pone.0352245.s005.docx]

| **S3 Table. This is the S3 Table Sensitivity Analysis included Mixed-Effect Model Analysis with Missing Imputation for Non-Responders (n=355) for PASC’s Impact on the Health Literacy Questionnaire Domains** | | | |
| --- | --- | --- | --- |
| Health Literacy Questionnaire Domains | Control/  Intervention  Mean  Difference | 95% CI | *P* Value |
| 1: Feeling understood and supported by healthcare providers | 0.012 | (-0.03-0.06) | 0.586 |
| 2: Having sufficient information to manage my health | -0.031 | (-0.07-0.00) | 0.082 |
| 3: Actively managing my health | 0.033 | (-0.00-0.07) | 0.056 |
| 4: Social support for health | 0.007 | (-0.03-0.04) | 0.700 |
| 5: Appraisal of health information | 0.006 | (-0.03-0.05) | 0.753 |
| 6: Ability to actively engage with healthcare providers | 0.012 | (-0.03-0.06) | 0.605 |
| 7: Navigating the healthcare system | -0.008 | (-0.05-0.04) | 0.731 |
| 8: Ability to find good health information | -0.031 | (-0.07-0.01) | 0.141 |
| 9: Understanding health information well enough to know what to do | 0.026 | (-0.01-0.07) | 0.186 |
| This is the S4 Table legend. Abbreviations: PASC=Patient Safety Checklist; CI=Confidence Interval  In the analysis control/intervention, time, and interaction were fixed factors, with clusters as random effects. The model was adjusted for age, sex, American Society of Anaesthesiology comorbidity risk classification scores (ASA), civil status, education, work status. The analysis was performed with missing imputation for education, work and civil status, domains, and the non-responders. | | | |
