# Supplementary material for: Effect of a patient-driven perioperative intervention on health literacy: A stepped-wedge cluster randomised sub-study
Source: PLoS One. 2026 Jun 24;21(6):e0352245. doi: 10.1371/journal.pone.0352245 (PMC13293430; doi:10.1371/journal.pone.0352245)
Supplement: S4 Table — (DOCX) [file pone.0352245.s006.docx]

| **S4 Table This is the S4 Table Cronbach’s Alpha Values and 95% Confidence Intervals for the** | | |
| --- | --- | --- |
| **Health Literacy Questionnaire Domains** | **Cronbach’ alpha** | **95 % CI** |
| 1. Feeling understood and supported by healthcare providers | 0.854 | (0.83-0.88) |
| 2. Having sufficient information to manage my health | 0.838 | (0.81-0.87) |
| 3. Actively managing my health | 0.850 | (0.82-0.87) |
| 4. Social support for health | 0.817 | (0.78-0.85) |
| 5. Appraisal of health information | 0.814 | (0.78-0.84) |
| 6. Ability to actively engage with healthcare providers | 0.862 | (0.84-0.89) |
| 7. Navigating the healthcare system | 0.873 | (0.85-0.89) |
| 8. Ability to find good health information | 0.833 | (0.80-0.86) |
| 9. Understanding health information well enough to know what to do | 0.725 | (0.67-0.77) |
| This is the S3 Table legend. Abbreviations: CI= Confidence Interval |  |  |
