# Supplementary material for: Effect of a patient-driven perioperative intervention on health literacy: A stepped-wedge cluster randomised sub-study
Source: PLoS One. 2026 Jun 24;21(6):e0352245. doi: 10.1371/journal.pone.0352245 (PMC13293430; doi:10.1371/journal.pone.0352245)
Supplement: Attachment — Submitted filename: Response to Reviewers.docx [file pone.0352245.s008.docx]

Dear Fatemeh Zarei, Academic Editor

Thank you very much for your rapid and through assessment of our manuscript: *“Impact of a patient safety checklist on health literacy outcomes –A sub-study of a stepped wedge cluster randomized controlled trial* “, and for the opportunity to submit a revised version. In what follows, we have restated the points raised by the editor/ reviewers (in italics) and detailed our response to each one of them- including the relevant amendments of the manuscript as required. The reviewer’s comments have been addressed chronologically.

Editors´comments:

*When submitting your revision, we need you to address these additional requirements.*

1. *Please ensure that your manuscript meets PLOS ONE¨s style requirements, including those for file naming. The PLOS ONE style templates can be found at*

[*https://journals.plos.org/plosone/s/file?id=wjVg/PLOSOne_formatting_sample_main_body.pdf*](https://journals.plos.org/plosone/s/file?id=wjVg/PLOSOne_formatting_sample_main_body.pdf) *and* [*https://journals.plos.org/plosone/s/file?id=ba62/PLOSOne_formatting_sample_title_authors_affiliations.pdf*](https://journals.plos.org/plosone/s/file?id=ba62/PLOSOne_formatting_sample_title_authors_affiliations.pdf)

Response to Editor

Thank you for your guidance regarding the PLOS ONE style requirements. We have carefully reviewed our manuscript and made changes in accordance with the provided guidelines. All modifications have been clearly marked with track changes in addition to a clean manuscript.

2. *We note that the grant information you provided in the “Funding Information” and “Financial Disclosure ‘sections do not match. When you resubmit, please ensure that you provide the correct grant numbers for the awards you received for your study in the “Funding Information” section.*

*If you would like to make changes to your financial disclosure, please include your updated statement in your cover letter.*

Response to Editor

Thank you for your comments regarding the grant information. We have reviewed and corrected the discrepancies between the “Funding Information” and “Financial Disclosure” sections.

Additionally, we have updated our financial disclosure statement accordingly and have included the revised statement in the cover letter as instructed:

“Dr. Arvid Steinar Haugen applied for this study’s funding, and the Norwegian Council of Nurses awarded the project with funding for a doctoral scholarship grant (ID number: 2 – 2022). This funding was administered by the Department of Anaesthesia and Intensive Care, Haukeland University Hospital, Bergen, Norway, and corresponding author Ann Kristin Sandsbakk Austarheim (AKSA) was employed as the PhD candidate in the study. The co-authors Kristin Harris (KH), Hilde Valen Wæhle (HVW), Anette Storesund (AS), Randi Julie Tangvik (RJT), and Arvid Steinar Haugen (ASH) were affiliated with the larger Stepped Wedge Cluster Randomized Controlled Trial of PASC, which was funded by the Norwegian Council of Research (ID number:320475). This funding was administered by the Department of Anaesthesia and Intensive Care, Haukeland University Hospital, Bergen, Norway. The funders had no role in study design, data collection and analysis, decision to publish, or preparation of the manuscript, and only provided financial support in the form of authors' salaries and research materials.”

The URLs to sponsors of this study:

1. [Norsk Sykepleierforbund - NSF](https://www.nsf.no/)
2. [Implementation of Patients Safety Checklist (PASC) in Surgery, a Stepped Wedge Cluster RCT - Effects on Patient and Implementation Outcomes - Prosjektbanken](https://prosjektbanken.forskningsradet.no/project/FORISS/320475?Kilde=FORISS&distribution=Ar&chart=bar&calcType=funding&Sprak=no&sortBy=date&sortOrder=desc&resultCount=30&offset=0&Fritekst=320475)

“The funders provided support in the form of salaries for authors [AKSA, KH, HVW, AS, RJT, and ASH], but did not have any additional role in the study design, data collection and analysis, decision to publish, or preparation of the manuscript. The specific roles of these authors are articulated in the ‘author contributions’ section.”

3. *Thank you for stating the following in the Competing Interests section:*

*“We have read the journal's policy, and the authors of this manuscript have the following competing interests: Arvid Steinar Haugen is member of the European Society of Anaesthesiologists’ and Intensive Care (ESAIC) Patient Safety and Quality Committee, representing the International Federation of Nurse Anaesthetists (IFNA). The other authors have declared that no competing interests exist.”*

*Please confirm that this does not alter your adherence to all PLOS ONE policies on sharing data and materials, by including the following statement: "This does not alter our adherence to PLOS ONE policies on sharing data and materials.” (as detailed online in our guide for authors* [*http://journals.plos.org/plosone/s/competing-interests*](http://journals.plos.org/plosone/s/competing-interests)*).  If there are restrictions on sharing of data and/or materials, please state these. Please note that we cannot proceed with consideration of your article until this information has been declared.*

*Please include your updated Competing Interests statement in your cover letter; we will change the online submission form on your behalf.*

Response to Editor

The updated Competing Interests statement has been included in our cover letter as requested:

“We have read the journal's policy, and the authors of this manuscript have the following competing interests: Arvid Steinar Haugen is member of the European Society of Anaesthesiologists’ and Intensive Care (ESAIC) Patient Safety and Quality Committee, representing the International Federation of Nurse Anaesthetists (IFNA). The other authors have declared that no competing interests exist. This does not alter our adherence to PLOS ONE policies on sharing data and materials.”

4. *Thank you for stating the following financial disclosure:*

*“**The Norwegian Council of Nurses granted a doctoral scholarship to AKSA, through the Department of Anaesthesia and Intensive Care, Haukeland University Hospital, Bergen, Norway, for this study. Kristin Harris (KH), Hilde Valen Wæhle (HVW), Anette Storesund (AS), Randi Julie Tangvik (RJT), and Arvid Steinar Haugen (ASH) are affiliated with the Stepped Wedge Cluster Randomized Controlled Trial of PASC, which is funded by the Norwegian Council of Research (ID:320475). The funders had no role in study design, data collection and analysis, decision to publish, or preparation of the manuscript.”*

*We note that one or more of the authors is affiliated with the funding organization, indicating the funder may have had some role in the design, data collection, analysis or preparation of your manuscript for publication; in other words, the funder played an indirect role through the participation of the co-authors. If the funding organization did not play a role in the study design, data collection and analysis, decision to publish, or preparation of the manuscript and only provided financial support in the form of authors' salaries and/or research materials, please do the following:*

*1. Review your statements relating to the author contributions and ensure you have specifically and accurately indicated the role(s) that these authors had in your study. These amendments should be made in the online form.*

Response to Editor:

We have in the online form made the following amendments:

“Dr. Arvid Steinar Haugen applied for this study’s funding, and the Norwegian Council of Nurses awarded the project with funding for a doctoral scholarship grant (ID number: 2 – 2022). This funding was administered by the Department of Anaesthesia and Intensive Care, Haukeland University Hospital, Bergen, Norway, and corresponding author Ann Kristin Sandsbakk Austarheim (AKSA) was employed as the PhD candidate in the study. The co-authors Kristin Harris (KH), Hilde Valen Wæhle (HVW), Anette Storesund (AS), Randi Julie Tangvik (RJT), and Arvid Steinar Haugen (ASH) were affiliated with the larger Stepped Wedge Cluster Randomized Controlled Trial of PASC, which was funded by the Norwegian Council of Research (ID number:320475). This funding was administered by the Department of Anaesthesia and Intensive Care, Haukeland University Hospital, Bergen, Norway. The funders had no role in study design, data collection and analysis, decision to publish, or preparation of the manuscript, and only provided financial support in the form of authors' salaries and research materials.”

The URLs to sponsors of this study:

1. [Norsk Sykepleierforbund - NSF](https://www.nsf.no/)
2. [Implementation of Patients Safety Checklist (PASC) in Surgery, a Stepped Wedge Cluster RCT - Effects on Patient and Implementation Outcomes - Prosjektbanken](https://prosjektbanken.forskningsradet.no/project/FORISS/320475?Kilde=FORISS&distribution=Ar&chart=bar&calcType=funding&Sprak=no&sortBy=date&sortOrder=desc&resultCount=30&offset=0&Fritekst=320475)

*2. Confirm in your cover letter that you agree with the following statement, and we will change the online submission form on your behalf:*

*“The funder provided support in the form of salaries for authors [insert relevant initials] but did not have any additional role in the study design, data collection and analysis, decision to publish, or preparation of the manuscript. The specific roles of these authors are articulated in the ‘author contributions’ section.”*

Response to Editor:

We have confirmed in our cover letter that we agree with the proposed statement and that the changes can be made in the online submission form on our behalf. We appreciate your assistance in making the changes directly in the online submission form. We also note that since there are two funders, we have changed "funder" to "funders" accordingly.

“The funders provided support in the form of salaries for authors [AKSA, KH, HVW, AS, RJT, and ASH], but did not have any additional role in the study design, data collection and analysis, decision to publish, or preparation of the manuscript. The specific roles of these authors are articulated in the ‘author contributions’ section.”

*5. In the online submission form you indicate that your data is not available for proprietary reasons and have provided a contact point for accessing this data. Please note that your current contact point is a co-author on this manuscript. According to our Data Policy, the contact point must not be an author on the manuscript and must be an institutional contact, ideally not an individual. Please revise your data statement to a non-author institutional point of contact, such as a data access or ethics committee, and send this to us via return email. Please also include contact information for the third-party organization, and please include the full citation of where the data can be found.*

Response to Editor

Thank you for this comment. In response, we have revised the data availability statement as requested. The updated statement has been submitted in the online form and sent to you via return email. The revised statement reads as follows:

“Data is not publicly available for proprietary reasons due to restrictions on data sharing according to the ethics committee REK WEST (2016-1102). For researchers whose proposed use of the data has been approved for research or educational purposes, anonymous data can be accessed after approval of a proposal and a signed data access agreement. Requests must be made to the Department of Anaesthesia and Intensive Care, Haukeland University Hospital, e-mail: [forskningksk@helse-bergen.no](mailto:forskningksk@helse-bergen.no).”

*6. If the reviewer comments include a recommendation to cite specific previously published works, please review and evaluate these publications to determine whether they are relevant and should be cited. There is no requirement to cite these works unless the editor has indicated otherwise.*

Response to Editor

Thank you for this advice. No additional citations have been added based on reviewer suggestions.

**Reviewers' comments:**

**Comments to the Authors**

1-4 (checklist points yes/no).

Author’s responses to Reviewers
1-4. The authors have no responses to these check points.

5.  Review Comments to the Authors

*Reviewer #1: As the statistical reviewer I will focus on methods and reporting*

*Major
1) The study had power problems because many participants scored at the very top of the health literacy scales, leaving little room to detect improvement, and the observed statistical power across outcomes was very low, meaning the study was unlikely to detect real differences even if they existed. Although the authors mention ceiling effects and note this may limit the ability to detect changes, they do not explicitly acknowledge that the study was under-powered overall, nor do they directly link the non‑significant findings to inadequate power. A driver of this was the very large, assumed effect size.*

Response to Reviewer#1

We thank the reviewer for highlighting the potential inadequate power of the study. As noted in the manuscript, a substantial proportion of participants scored at or near the upper limit of the health literacy scales, which may have reduced variability and limited the sensitivity to detect changes. While ceiling effects may have reduced the effective ability to detect change in this sample, we think this represents a measurement and sample characteristic limitation rather than inadequate a priori statistical power. We do acknowledge that the post hoc power observation indicated a low sample size and that variations in non-responder characteristics across the clusters, especially for non-responders could have influenced on the required sample size. Hence, we have included this possibility in the limitation section on page 17, lines 333-342:

“Several limitations should be considered when interpreting the results. The overall health literacy domain levels demonstrated ceiling effects in both control and intervention groups, which may have limited the ability to detect changes. Even though a priory sample size was estimated, it is possible that a higher number of respondents and participants could have increased the study’s overall ability to detect changes. Especially since younger and healthier participants responded less than older patients. In the general Norwegian population, younger people between 18-24 years are reported to have challenges with interacting with healthcare professionals [30]. Based on this underlying difference between responders and non-responders, we cannot exclude the risk that the statistical power was limited and could have increased with a larger sample.”

*Reviewer#1*

*2) report uncertainty in Cronbach's alpha, e.g. bootstrap.*

Response to Reviewer#1

Thank you for this comment. We found that the bootstrapping option for the Cronbach alphas were not available within the Reliability Analysis procedure in SPSS Statistics v. 31.0 However, we were able to produce equal confidence interval estimates by using the Intraclass correlation coefficient (ICC), absolute agreement, and reporting the average measures.

In Statistical Analysis we have added this information on page 9, lines 212-215:

“To account for variability in Cronbach’s alpha domain measures, we calculated 95% confidence intervals (CI’s) using the intraclass correlation coefficient (ICC) with absolute agreement, and the average measures.”

Under results, we have listed the results on page 14, lines 262-264, and added the 95% CI in the S3 Table:

“The Cronbach’s alpha values ranged from 0.725 to 0.873 across the nine domains and are reported with corresponding 95% CI’s in the S4 Table.”

*Reviewer#1*

3*) no information as to how missing data were handled. all data were not complete I presume, so why weren't multiple imputation approaches used?*

Response to Reviewer#1

Thank you very much for this comment. We agree that a more detailed description was warranted. We observed few missing values in the data set; the highest missing proportion was for patient characteristics as Education (n=20). And for the items in the nine domains, there were few missing values. The missing data was mainly completely at random (MCAR) for eight domains or random (MAR) for one domain. As the missing values did not exceed the original limits of the sample size calculations for our regression analysis, we did not apply any imputations for the missing values. However, based on the possibility for being under-powered we have now made new analysis, with multiple imputation in SPSS, both for Table 2 and Table 3. We have rephrased our text in Statistical Analyses, page 9 lines 203-211:

“Patterns of missing data were controlled for patient characteristics and the HLQ domain items. Baseline characteristics of responders and non‑responders were compared to assess potential attrition biases. Responders and non-responder differences were examined for the variables age, sex, comorbidities (ASA), and clusters by using descriptive analyses. To avoid risk of losing power in the analyses, the regression analysis was performed with imputed data for education, civil status, work status, and the HLQ domains. Imputation was generated by 10 imputations with an automatically imputation method based on the missing pattern in the data. For sensitivity analysis we imputed non-responders’ missing outcome values based on the observed responder outcomes.”

Further, we have added the frequencies of missing data in Table 1 (page 10)

*Reviewer#1*

*4) The analyses treated clusters as fixed covariates in a simple GLM rather than as random effects, which means the stepped‑wedge structure and within‑cluster correlation were not properly modelled. This is a methodological limitation because stepped‑wedge cluster trials typically require mixed‑effects models with random cluster effects to produce valid inferences. Can the authors expand on their choice?*

Response to Reviewer#1

Again, thank you for this valuable comment regarding the modelling of cluster effects in stepped wedge cluster trials. We agree that treating clusters as fixed covariates in a simple GLM does not properly account for the within-cluster correlation, especially when the goal is to generalize our findings. Based on the reviewer’s concern, we have conducted new analyses in SPSS using the mixed-effects model analysis with random cluster effects. We treated the control/intervention variable and the time as fixed factors, and cluster as a random effects, while age, sex, ASA (comorbidity), civil status, education and work status were covariates. Since time and control/intervention may have some overlap in Stepped Wedge designs, we performed an additional analysis, using the same model and adding an interaction variable for control/interaction and time. Accordingly, we revised the statistical methods section to describe this change on page 8-9, lines 193-200:

“Analysis of Variance (ANOVA) was used to compare mean score differences between control and intervention groups on the HLQ domains. Further, we used General Linear Mixed Model to assess the impact PASC had on the nine HLQ domains. Each HLQ domain was treated as dependent variables, with control/intervention and time as fixed factors. The clusters were modelled as random effects to produce valid inferences for the mixed-effects model, with patient characteristics were treated as covariates. Additionally, we investigated if the outcomes were influenced by interactions between control/intervention and time by adding this interaction to the factor model.”

Further, Table 3 has been amended to account for this new analysis (page 13). We have removed the ‘Observed power’ column as it: 1) was a post hoc analysis, and 2) was not available in the mixed-effects model analysis.

Under Results, page 14, lines 252-253 we have described the results from the additional analysis with interaction:

“Overall results from the mixed-effects regression analysis showed no statistically significant differences between the groups (Table 3).”

*Reviewer#1*

*5) Bonferroni corrections further reduce power, dramatically. this was not considered in the power calculations. effectively it compounds the problem severely.*

Response to Reviewer#1

Thank you for this observation. We have re-analysed the data without Bonferroni corrections.

*Reviewer#1*

*6) the very high non response rate, and the risk of bias with younger/healthier patients less likely to respond is another problem. Arguably, sensitivity analyses with multiple imputations and making assumptions for non-responders would provide some more information.*

Response to Reviewer#1

Thank you for this suggestion. We agree that sensitivity analyses with multiple imputations could add more information on the non-responders even though this analysis will rest on the responders observed domain scores. As mentioned above, we performed a multiple imputation, with 10 imputations for the entire data set (n=700). We performed mixed model analysis with the imputed data set (pooled), and we observed only very small changes compared to previous findings, indicating robustness of the analyses. We have reported this on page 14, lines 259-260:

“Sensitivity analysis with imputed data including non-responders showed very small changes and indicated robustness of the analyses (S3 Table).”

*Reviewer#1*

*Minor
1) univariable (not univariate) regressions are mentioned but multivariable regressions are reported?*

Response to Reviewer#1:

Thank you for your comment. We did perform univariate regression analyses as part of our assessment of the impact of the intervention on the nine Health Literacy Questionnaire (HLQ) domains. Each HLQ domain was treated as a dependent variable in the univariate regression model. Since the HLQ domains are stand-alone domains, we could not do a multivariable regression. To clarify this, we have detailed this more in the Statistical analysis section, page 8, line 195-197:

“Each HLQ domain was treated as dependent variables, with control/intervention and time as fixed factors.”

*Reviewer #2*

*Title Mentioning the tools in title is not common the title emphasizes the ("checklist") rather than the mechanism or outcome. In the context of a null result (which we see in the abstract), this title is particularly problematic. Its suggestion: Patient Activation and Preoperative Health Literacy: Findings from a Stepped-Wedge Randomized Trial OR Title: Change to remove "checklist" and adopt a more classic structure (e.g., "Effect of a Patient-Driven Preoperative Intervention on Health Literacy: A Stepped-Wedge Cluster Randomized Sub-Study").*

Response to reviewer#2:

Thank you very much for your important comments regarding the manuscript title. We appreciate your important suggestion. We have amended the title to: "Effect of a Patient-Driven Perioperative Intervention on Health Literacy: A Stepped-Wedge Cluster Randomized Sub-Study”.

This change is presented on the title page, page 1, lines 1-2.

*Reviewer#2*

*Abstract This is a strong opening in terms of scientific method (stating the hypothesis upfront). However, as a standalone abstract for a paper with null results, it sets a tone of "failure" that needs to be mitigated in the conclusion. No revision needed here.*

Response to Reviewer#2

Thank you for your positive feedback on the abstract’s clear statement of the hypothesis. In response to your comment regarding the tone of the conclusion, we have revised the conclusion section to better reflect the value of the findings despite null results.

*Reviewer#2*

*Typo: "delivered preoperatively (≤ eight weeks." -> Missing closing parenthesis: (≤ eight weeks).*

Response to Reviewer#2

Thank you very much for pointing out the typo. We have corrected this on page 2, line 48, by adding the closing parenthesis.

*Reviewer#2*

*“In each cluster, 50 patients were randomly drawn from 100 eligible patients, a total of 350 in each group. This is a confusing description of sampling. If it's a sub-study of an RCT, were the 700 patients the entire trial cohort for this sub-study? Or were 700 drawn from a larger pool? The phrase "randomly drawn from 100 eligible" suggests a random selection process within clusters.”*

Response to Reviewer#2

Thank you for this comment. We agree that the description is unclear. This study was a sub-study of a larger randomized controlled trial (RCT). It is correct that the 700 patients in this sub-study were selected from a larger pool within the trial. From the larger trial, 100 eligible patients were identified in each cluster in control and in intervention group. A random selection of 50 patients was performed within each cluster and group. We agree that the original wording was unclear and have now revised the manuscript to clarify the sampling process.

We have included justifications in the Abstract, on page 2, lines 40-44:

“The patients were included from a pool within the cluster trial. In each of these seven clusters, 50 patients were randomly selected from 100 eligible patients from both the control and the intervention group using a computer-generated randomisation procedure. This resulted in a total sample of 700 patients: 350 in each group, response rate 49.3 %.”

*Reviewer#2*

*Results: Condense the 9 statistical lines into a single, clear summary sentence.*

Response to Reviewer#2

Thank you for your valuable feedback. We have now condensed the main findings into the concise summary sentence as suggested.

This has been updated and is presented on page 2, lines 49-51:

“No significant differences were observed in mean scores across the nine health literacy outcome domains between the control and intervention groups.”

*Reviewer#2*

*Conclusion "The use of a patient-driven safety checklist did not lead to measurable improvements across health literacy questionnaire domains, and the hypothesis was not supported. The questionnaire tool had signs of ceiling effects, hence the checklist’s influence on patients’ health literacy remains unclear."
"The hypothesis was not supported." vs "The checklist's influence... remains unclear."
You cannot have it both ways in a single abstract. Either the intervention failed (null result) OR the measurement tool failed (inconclusive result).
Suggestion for Rewrite: "**In this stepped-wedge cluster trial, a preoperative patient safety checklist did not improve health literacy scores compared to standard care. However, the high baseline scores (ceiling effect) and a 49% response rate limit definitive interpretation of the intervention's true effect. Further research should focus on patient populations with lower baseline health literacy or utilize more sensitive measurement tools."*

Response to Reviewer#2

We thank you for this very important feedback. We agree to your comment and have amended this in the abstract, page 2, lines 51-56:

“In this stepped wedge cluster trial, a preoperative patient safety checklist did not improve health literacy scores compared to standard care. Nevertheless, an interaction effect with time for the domain ‘Actively managing my health’, may indicate that the patient-driven checklist could support elective surgical patients’ health management. This finding aligns with qualitative feedback and warrants further investigation using larger samples and more sensitive and context-specific measurement tools.”

*Reviewer#2*

*Introduction
• Change "reveal" to "reveals" or "revealed".*

Response to Reviewer#2

Thank you for pointing out the grammatical correction. We have updated the manuscript accordingly, changing "reveal" to "reveals".

The correction is made on page 3, line 71:

“Despite this need, a recent literature review reveals a scarcity of health literacy interventions tailored to surgical patients [9].”

*• Add a sentence defining the specific domain of health literacy targeted.*

Response to reviewer#2

Thank you for the suggestion. Since our study explores all domains of the Health Literacy Questionnaire (HLQ), we have added a sentence in the manuscript clearly stating that all HLQ domains were targeted.

This sentence has been added on page 3, lines 90-92:

“The study assessed all nine distinct domains of health literacy as defined by the Health Literacy Questionnaire (HLQ), providing a comprehensive evaluation of patients’ health literacy skills and experiences [14].”

*Reviewer#2*

*• Verify and potentially update Reference [9] regarding recent interventions.*

Response to Reviewer#2

Thank you for the suggestion. The corresponding author performed an updated literature search together with a medical librarian in February 2026. An updated search identified more recent single studies, but they did not alter the relevance or applicability of reference [9]. Therefore, we decided to make no changes for this reference.

*Reviewer#2*

*• Provide one concrete example of a PASC checklist item.*

Response to Reviewer#2

We welcome this suggestion. A concrete example of a PASC item is provided in the introduction. We have added one of the items as an example, on page 3-4, lines 80-83:

“A concrete PASC item is: "Do you use blood-thinning medication?" This item is followed by instructions regarding medication management and advice to contact the surgical ward if clarification is needed. Such items are designed to support patients in identifying and acting upon important perioperative safety issues [10].”

*Reviewer#2*

*• Add a brief note acknowledging the measurement challenge in this specific cohort.*

Response to Reviewer#2

Thank you again for this proposal. We agree and have amended the text and added a brief sentence to acknowledge this challenge on page 3, lines 72-74:

“It may especially be challenging to measure and reliably interpret outcome in patients undergoing surgical interventions due to heterogeneity in patient and procedures characteristics.”

*Reviewer#2*

*Method
• Timeline Discrepancy: 2022 vs 2023 start date
• Correct the date in Abstract or Methods to match source data.*

Response to Reviewer#2

Thank you for pointing out the discrepancy regarding the timeline. The correct start date is 1 March 2022, and this has now been updated accordingly in the method section on page 4, line 97.

*Reviewer#2*

*Design Lack of detail on stepped wedge steps/duration Add 1-2 sentences clarifying the number and length of steps.*

Response to Reviewer#2

Thank you for valuable comment. We have added 2 sentences to clarify the stepped-wedge design, including the number of steps and the duration of clusters.

These two sentences are presented on page 4-5, lines 103-108:

“In the control group all clusters performed standard surgical care. Following a randomized order the first cluster then switched to intervention group where patients used the PASC intervention. The other clusters still providing standard surgical care. After the next two months, the second cluster switched to intervention group, and this went on until all clusters had crossed over from control to intervention groups. For the sub-study this included a 14-months period.”

*Reviewer#2*

*Sampling
• "Randomly drawn 50 from 100" - Justification missing Explain why random sub-sampling was used (resource/logistical constraint).*

Response to Reviewer#2

Thank you for highlighting this important point. Based on the sample size calculation, a random sub-sampling of 50 of 100 eligible patients per cluster was performed, both in the control and intervention groups. Primarily, this was done to minimize unnecessary patient burden and exposure to research questionnaires, and secondly for logistical and resource constraints.

The justification for the random sub-sampling approach is explained on page 5, lines 115–117:

“This random sub-sampling approach was primarily done to minimize unnecessary patient burden and exposure to research questionnaires, and secondly for logistical and resource constraints.”

*Reviewer#2*

*• ITT Analysis Unclear handling of missing data for 355 non-responders Specify: Complete Case Analysis or Imputation method*

Response to Reviewer#2

Thank you for this remark. We agree that the term ITT was inaccurate and that we performed a modified ITT analysis, as we did not have outcome data from non-responders. Our text on this was unclear and have made the following changes in the Method section on page 9, lines 207-211:

“To avoid risk of losing power in the analyses, the regression analysis was performed with imputed data for education, civil status, work status, and the HLQ domains. Imputation was generated by 10 imputations with an automatically imputation method based on the missing pattern in the data. For sensitivity analysis we imputed non-responders’ missing outcome values based on the observed responder outcomes.”

*Reviewer#2*

*Outcome Measure No mention of known ceiling effect in this population Add note about ceiling effect risk in Primary Outcome section.*

Response to Reviewer#2

Thank you for your comment. We agree and have now added a note in the Primary Outcome section acknowledging the potential risk of ceiling effects. As evidence regarding ceiling effects of the HLQ in this population is limited, this was addressed as a theoretical consideration and considered in the interpretation of the results. We added a few sentences on this under Primary Outcomes, on page 7, lines 164-168:

“Although the HLQ has demonstrated good psychometric properties across diverse populations [14, 20], there remains a theoretical possibility of ceiling effects. Such effects have been reported in other HLQ studies [21, 22]. Given the limited existing evidence on possible ceiling effects of the HLQ in his specific population, this aspect needs to be analysed.”

*Reviewer#2*

• *Typos Multiple minor spelling/formatting errors*

Response to Reviewer#2

Thank you for pointing out the minor spelling and formatting errors. We have carefully reviewed the manuscript and both spelling and formatting errors in the Method section.

*Reviewer#2*

*Result
The Non-Responder Analysis is Flawed in Logic
"Non-responders were younger than responders (mean age 54.1 vs. 62.0 years; P = 0.025) and more likely to have ASA I classification (60.4% vs. 39.6%)."
The text states "Non-responders were... more likely to have ASA I classification."
But ASA I means healthy patient. The data says 60.4% of non-responders were ASA I vs 39.6% of responders.
So Healthier, younger patients were LESS likely to respond.
This is the opposite of typical survey bias. Usually, sicker patients are too tired to respond. Here, healthier patients (who likely have higher health literacy) ignored the survey.*

*Impact on Results: If the healthiest/highest literacy patients dropped out of the intervention group, the intervention effect might be underestimated. The authors MUST discuss this in the Limitations section (currently they only mention younger/healthier responded less, but don't connect the dots to health literacy bias). This is a major omission.*

Response to Reviewer#2

Thank you for this important and insightful comment. We agree that our original description did not sufficiently articulate the logical implications of the non‑responder analysis. As correctly noted, non‑responders were younger and healthier (ASA I), indicating that healthier patients were less likely to respond, which differs from the more common pattern of attrition among sicker individuals.

We have rephrased the result description on page 14, lines 257-258:

“Healthier, younger patients were less likely to respond, with mean age 54.1 vs. 62.0 years; P = 0.025, and ASA I classification at 60.4% vs. 39.6%.”

We have also expanded the Limitations section to explicitly discuss how this differential non‑response may have biased the results. Given that younger and healthier patients are also likely to have higher levels of baseline health literacy, their under‑representation at follow‑up may have resulted in an underestimation of the true intervention effect on health literacy outcomes. This issue has now been clearly acknowledged and discussed on page 17, lines 333-345:

“Several limitations should be considered when interpreting the results. The overall health literacy domain levels demonstrated ceiling effects in both control and intervention groups, which may have limited the ability to detect changes. Even though a priory sample size was estimated, it is possible that a higher number of respondents and participants could have increased the study’s overall ability to detect changes. Especially since younger and healthier participants responded less than older patients. In the general Norwegian population, younger people between 18-24 years are reported to have challenges with interacting with healthcare professionals [30]. Based on this underlying difference between responders and non-responders, we cannot exclude the risk that the statistical power was limited and could have increased with a larger sample. However, even if sensitivity analysis based on responder outcomes could not completely account for the large proportion of non-responses, it showed that with imputed data for non-responders there were no significant changes, indicating robustness in the analysis.”

*Reviewer#2*

*Discussion

"These findings suggest that there is no clear association... raising two possible explanations: either PASC did not directly affect health literacy domains, or the HLQ instrument lacked the sensitivity..."

This is the exact same sentence logic as the Abstract Conclusion. While consistency is good, the Discussion should expand on this, not repeat it verbatim. The first paragraph of a Discussion should summarize the main finding and then immediately pivot to interpretation.
Redundancy. Please rewrite.*

Response to Reviewer#2

Thank you for this comment. We acknowledge that it is necessary to rephrase the opening of the Discussion section. To address this, we have amended the text on page 14-15, lines 266-282:

“To our knowledge, this is the first stepped‑wedge trial to evaluate the impact of a patient safety checklist combined with standard surgical information on elective surgical patients’ health literacy using the HLQ. The intervention was not associated with statistically significant changes in HLQ domain scores, and the overall study hypothesis was therefore not supported. The mixed-effects model with including cluster as random effects, adjusting for relevant covariates did not alter these findings. However, pairwise comparisons suggested that domain 3 (Actively managing my health) was significantly improved, when accounting for interaction between time and control/intervention groups. This finding may indicate that a larger sample could identify possible impact of the patient-driven checklist. This was supported by findings in a recent published qualitative study, where using the PASC raised their awareness on safety issues, and led to actions for ensuring medication control, optimization of health, and increasing their interactions with healthcare personnel [11]. Rather than indicating a lack of relevance of the intervention per se, the overall results are best interpreted considering methodological and contextual factors. In particular, the high baseline health literacy observed in this Norwegian cohort, together with ceiling effects in several HLQ domains, suggests that the instrument may have had limited sensitivity to detect modest or context‑specific changes attributable to the PASC intervention.”

*Reviewer#2*

*The "Trend" Signal in Domain 2 is Ignored in the Discussion
Discussion mentions ceiling effects and qualitative findings but never addresses the near-significant 0.90 Beta in Domain 2…*

Response to Reviewer#2

Thank you for your insightful comment regarding the "Trend" signal in Domain 2. Following Reviewer #1's recommendation, we revised our analyses to use a mixed-effects model approach with clusters as random effect, which better accounts for the stepped wedge design and within-cluster correlation. Due to this change in the analysis, the trend we saw previously was not so prominent. However, we identified possible impact on domain 3 (Actively managing my health) after adding interaction between time and control/intervention in the regression model.

This finding has been included in Abstract page 2, lines 53-55, Method section on page 8, lines 192-198, Result section page 14, lines 253-256, Discussion section on page 14, lines 271-273, and in the Conclusion on page 18, lines 360-362.

*Reviewer#2*

*[PASC is considered highly operational, context specific and oriented toward patient safety, and oriented toward patient safety] “oriented toward patient safety” duplicated in one sentence delete one.*

Response to Reviewer#2

Thank you for pointing out the duplication in the sentence. We have corrected the text by removing the repeated phrase "oriented toward patient safety".

The repeated phrase is removed on page 15, line 284.

*Reviewer#2*

*Conclusion

The conclusion should synthesize the three key takeaways from the Discussion:
1. Primary Finding: No significant difference in HLQ scores.
2. Methodological Insight: Ceiling effects in this high-functioning Norwegian cohort likely masked any true effect; the HLQ may be inappropriate for this specific intervention evaluation.
3. Signal of Interest: The trend toward improved information management (Domain 2) aligns with qualitative feedback and warrants further investigation using more sensitive, context-specific tools.*

Response to Reviewer#2

Thank you for your valuable feedback. We have revised the conclusion to clearly synthesize the three key points from the discussion you suggested and based on the up-dated analysis we have included the interaction effect finding. The revised version is presented on page 18, lines 357-364:

“The overall findings showed no significant difference in health literacy domain scores following the intervention. The presence of ceiling effects in this relatively high-functioning Norwegian patient population may have masked a true effect, suggesting that the HLQ instrument may be less suitable for evaluating this specific intervention. Nevertheless, an interaction effect with time for the domain ‘Actively managing my health’, may indicate that the patient-driven checklist could support elective surgical patients’ health management. This finding aligns with qualitative feedback and warrants further investigation using larger samples and more sensitive and context-specific measurement tools.”

Finally, we would like to thank the reviewers for their thorough and thoughtful comments, which were very helpful, pointing out correct statistical analyses, and how to present this study.
