# Supplementary material for: Effect of a patient-driven perioperative intervention on health literacy: A stepped-wedge cluster randomised sub-study
Source: PLoS One. 2026 Jun 24;21(6):e0352245. doi: 10.1371/journal.pone.0352245 (PMC13293430; doi:10.1371/journal.pone.0352245)
Supplement: Peer review history

# Peer review: Decision Letter 0

**DOI:** 10.1371/journal.pone.0352245.r001

**Authors:** Fatemeh Zarei

15 Apr 2026

PONE-D-26-02760

Impact of a patient safety checklist on health literacy outcomes – A sub-study of a stepped wedge cluster randomized controlled trial

PLOS One

Dear Dr. Austarheim,

Thank you for submitting your manuscript to PLOS ONE. After careful consideration, we feel that it has merit but does not fully meet PLOS ONE’s publication criteria as it currently stands. Therefore, we invite you to submit a revised version of the manuscript that addresses the points raised during the review process.

Please submit your revised manuscript by May 30 2026 11:59PM. If you will need more time than this to complete your revisions, please reply to this message or contact the journal office at plosone@plos.org. When you're ready to submit your revision, log on to https://www.editorialmanager.com/pone/ and select the 'Submissions Needing Revision' folder to locate your manuscript file.

Please include the following items when submitting your revised manuscript:

- A letter that responds to each point raised by the academic editor and reviewer(s). You should upload this letter as a separate file labeled 'Response to Reviewers'.
- A marked-up copy of your manuscript that highlights changes made to the original version. You should upload this as a separate file labeled 'Revised Manuscript with Track Changes'.
- An unmarked version of your revised paper without tracked changes. You should upload this as a separate file labeled 'Manuscript'.

If you would like to make changes to your financial disclosure, please include your updated statement in your cover letter. Guidelines for resubmitting your figure files are available below the reviewer comments at the end of this letter.

If applicable, we recommend that you deposit your laboratory protocols in protocols.io to enhance the reproducibility of your results. Protocols.io assigns your protocol its own identifier (DOI) so that it can be cited independently in the future. For instructions see: https://journals.plos.org/plosone/s/submission-guidelines#loc-laboratory-protocols. Additionally, PLOS ONE offers an option for publishing peer-reviewed Lab Protocol articles, which describe protocols hosted on protocols.io. Read more information on sharing protocols at https://plos.org/protocols?utm_medium=editorial-email&utm_source=authorletters&utm_campaign=protocols.

As the corresponding author, your ORCID iD is verified in the submission system and will appear in the published article. PLOS supports the use of ORCID, and we encourage all coauthors to register for an ORCID iD and use it as well. Please encourage your coauthors to verify their ORCID iD within the submission system before final acceptance, as unverified ORCID iDs will not appear in the published article. *Only*  the individual author can complete the verification step; PLOS staff *cannot*  verify ORCID iDs on behalf of authors.

We look forward to receiving your revised manuscript.

Kind regards,

Fatemeh Zarei, PhD

Academic Editor

PLOS One

Journal Requirements:

When submitting your revision, we need you to address these additional requirements.

1. Please ensure that your manuscript meets PLOS ONE's style requirements, including those for file naming. The PLOS ONE style templates can be found at

https://journals.plos.org/plosone/s/file?id=wjVg/PLOSOne_formatting_sample_main_body.pdf and

https://journals.plos.org/plosone/s/file?id=ba62/PLOSOne_formatting_sample_title_authors_affiliations.pdf

2. We note that the grant information you provided in the ‘Funding Information’ and ‘Financial Disclosure’ sections do not match.

When you resubmit, please ensure that you provide the correct grant numbers for the awards you received for your study in the ‘Funding Information’ section.

3. Thank you for stating the following in the Competing Interests section:

“We have read the journal's policy and the authors of this manuscript have the following competing interests: Arvid Steinar Haugen is member of  the European Society of Anaesthesiologists’ and Intensive Care (ESAIC) Patient Safety and Quality Committee, representing the International Federation of Nurse Anaesthetists (IFNA). The other authors have declared that no competing interests exist.”

Please confirm that this does not alter your adherence to all PLOS ONE policies on sharing data and materials, by including the following statement: "This does not alter our adherence to  PLOS ONE policies on sharing data and materials.” (as detailed online in our guide for authors http://journals.plos.org/plosone/s/competing-interests).  If there are restrictions on sharing of data and/or materials, please state these. Please note that we cannot proceed with consideration of your article until this information has been declared.

Please include your updated Competing Interests statement in your cover letter; we will change the online submission form on your behalf.

4. Thank you for stating the following financial disclosure:

“The Norwegian Council of Nurses granted a doctoral scholarship to AKSA, through the Department of Anesthesia and Intensive Care, Haukeland University Hospital, Bergen, Norway, for this study. Kristin Harris (KH), Hilde Valen Wæhle (HVW), Anette Storesund (AS), Randi Julie Tangvik (RJT), and Arvid Steinar Haugen (ASH) are affiliated with the Stepped Wedge Cluster Randomized Controlled Trial of PASC, which is funded by the Norwegian Council of Research (ID:320475). The funders had no role in study design, data collection and analysis, decision to publish, or preparation of the manuscript.”

We note that one or more of the authors is affiliated with the funding organization, indicating the funder may have had some role in the design, data collection, analysis or preparation of your manuscript for publication; in other words, the funder played an indirect role through the participation of the co-authors. If the funding organization did not play a role in the study design, data collection and analysis, decision to publish, or preparation of the manuscript and only provided financial support in the form of authors' salaries and/or research materials, please do the following:

1. Review your statements relating to the author contributions, and ensure you have specifically and accurately indicated the role(s) that these authors had in your study. These amendments should be made in the online form.

2. Confirm in your cover letter that you agree with the following statement, and we will change the online submission form on your behalf:

“The funder provided support in the form of salaries for authors [insert relevant initials], but did not have any additional role in the study design, data collection and analysis, decision to publish, or preparation of the manuscript. The specific roles of these authors are articulated in the ‘author contributions’ section.

5. In the online submission form you indicate that your data is not available for proprietary reasons and have provided a contact point for accessing this data. Please note that your current contact point is a co-author on this manuscript. According to our Data Policy, the contact point must not be an author on the manuscript and must be an institutional contact, ideally not an individual. Please revise your data statement to a non-author institutional point of contact, such as a data access or ethics committee, and send this to us via return email. Please also include contact information for the third party organization, and please include the full citation of where the data can be found.

6. If the reviewer comments include a recommendation to cite specific previously published works, please review and evaluate these publications to determine whether they are relevant and should be cited. There is no requirement to cite these works unless the editor has indicated otherwise.

[Note: HTML markup is below. Please do not edit.]

Reviewers' comments:

Reviewer's Responses to Questions

**Comments to the Author**

1. Is the manuscript technically sound, and do the data support the conclusions?

The manuscript must describe a technically sound piece of scientific research with data that supports the conclusions. Experiments must have been conducted rigorously, with appropriate controls, replication, and sample sizes. The conclusions must be drawn appropriately based on the data presented.

Reviewer #1: Yes

Reviewer #2: Yes

**********

2. Has the statistical analysis been performed appropriately and rigorously?

Reviewer #1: Yes

Reviewer #2: No

**********

3. Have the authors made all data underlying the findings in their manuscript fully available?

The PLOS Data policy requires authors to make all data underlying the findings described in their manuscript fully available without restriction, with rare exception (please refer to the Data Availability Statement in the manuscript PDF file). The data should be provided as part of the manuscript or its supporting information, or deposited to a public repository. For example, in addition to summary statistics, the data points behind means, medians and variance measures should be available. If there are restrictions on publicly sharing data—e.g. participant privacy or use of data from a third party—those must be specified.

Reviewer #1: Yes

Reviewer #2: Yes

**********

4. Is the manuscript presented in an intelligible fashion and written in standard English?

PLOS ONE does not copyedit accepted manuscripts, so the language in submitted articles must be clear, correct, and unambiguous. Any typographical or grammatical errors should be corrected at revision, so please note any specific errors here.

Reviewer #1: Yes

Reviewer #2: Yes

**********

5. Review Comments to the Author

Please use the space provided to explain your answers to the questions above. You may also include additional comments for the author, including concerns about dual publication, research ethics, or publication ethics. (Please upload your review as an attachment if it exceeds 20,000 characters)

Reviewer #1: As the statistical reviewer I will focus on methods and reporting

Major

1) The study had power problems because many participants scored at the very top of the health literacy scales, leaving little room to detect improvement, and the observed statistical power across outcomes was very low, meaning the study was unlikely to detect real differences even if they existed. Although the authors mention ceiling effects and note this may limit the ability to detect changes, they do not explicitly acknowledge that the study was under-powered overall, nor do they directly link the non‑significant findings to inadequate power. A driver of this was the very large assumed effect size.

2) report uncertainty in Cronbach's alpha, e.g. bootstrap.

3) no information as to how missing data were handled. all data were not complete I presume, so why weren't multiple imputation approaches used?

4) The analyses treated clusters as fixed covariates in a simple GLM rather than as random effects, which means the stepped‑wedge structure and within‑cluster correlation were not properly modelled. This is a methodological limitation because stepped‑wedge cluster trials typically require mixed‑effects models with random cluster effects to produce valid inferences. Can the authors expand on their choice?

5) Bonferroni corrections further reduce power, dramatically. this was not considered in the power calculations. effectively it compounds the problem severely.

6) the very high non response rate, and the risk of bias with younger/healthier patients less likely to respond is another problem. Arguably, sensitivivity analyses with multiple imputations and making assumptions for non-responders would provide some more information.

Minor

1) univariable (not univariate) regressions are mentioned but multivariable regressions are reported?

Reviewer #2: Title

Mentioning the tools in title is not common The title emphasizes the ("checklist") rather than the mechanism or outcome. In the context of a null result (which we see in the abstract), this title is particularly problematic. Its suggestion : Patient Activation and Preoperative Health Literacy: Findings from a Stepped-Wedge Randomized Trial OR Title: Change to remove "checklist" and adopt a more classic structure (e.g., "Effect of a Patient-Driven Preoperative Intervention on Health Literacy: A Stepped-Wedge Cluster Randomized Sub-Study").

Abstract

This is a strong opening in terms of scientific method (stating the hypothesis upfront). However, as a standalone abstract for a paper with null results, it sets a tone of "failure" that needs to be mitigated in the conclusion. No revision needed here

Typo: "delivered preoperatively (≤ eight weeks." -> Missing closing parenthesis: (≤ eight weeks)

"In each cluster, 50 patients were randomly drawn from 100 eligible patients, a total of 350 in each group."

This is a confusing description of sampling. If it's a sub-study of an RCT, were the 700 patients the entire trial cohort for this sub-study? Or were 700 drawn from a larger pool? The phrase "randomly drawn from 100 eligible" suggests a random selection process within clusters.

Results: Condense the 9 statistical lines into a single, clear summary sentence.

Conclusion

"The use of a patient-driven safety checklist did not lead to measurable improvements across health literacy questionnaire domains, and the hypothesis was not supported. The questionnaire tool had signs of ceiling effects, hence the checklist’s influence on patients’ health literacy remains unclear."

"The hypothesis was not supported." vs "The checklist's influence... remains unclear."

You cannot have it both ways in a single abstract. Either the intervention failed (null result) OR the measurement tool failed (inconclusive result).

Suggestion for Rewrite: "In this stepped-wedge cluster trial, a preoperative patient safety checklist did not improve health literacy scores compared to standard care. However, the high baseline scores (ceiling effect) and a 49% response rate limit definitive interpretation of the intervention's true effect. Further research should focus on patient populations with lower baseline health literacy or utilize more sensitive measurement tools."

Introduction

• Change "reveal" to "reveals" or "revealed".

• Add a sentence defining the specific domain of health literacy targeted.

• Verify and potentially update Reference [9] regarding recent interventions.

• Provide one concrete example of a PASC checklist item.

• Add a brief note acknowledging the measurement challenge in this specific cohort.

method

• Timeline Discrepancy: 2022 vs 2023 start date

• Correct the date in Abstract or Methods to match source data.

• Design Lack of detail on stepped wedge steps/duration Add 1-2 sentences clarifying the number and length of steps.

Sampling

• "Randomly drawn 50 from 100" - Justification missing Explain why random sub-sampling was used (resource/logistical constraint).

• ITT Analysis Unclear handling of missing data for 355 non-responders Specify: Complete Case Analysis or Imputation method

• Outcome Measure No mention of known ceiling effect in this population Add note about ceiling effect risk in Primary Outcome section.

• Typos Multiple minor spelling/formatting errors

Result

The Non-Responder Analysis is Flawed in Logic

"Non-responders were younger than responders (mean age 54.1 vs. 62.0 years; P = 0.025) and more likely to have ASA I classification (60.4% vs. 39.6%)."

The text states "Non-responders were... more likely to have ASA I classification."

But ASA I means healthy patient. The data says 60.4% of non-responders were ASA I vs 39.6% of responders.

So Healthier, younger patients were LESS likely to respond.

This is the opposite of typical survey bias. Usually, sicker patients are too tired to respond. Here, healthier patients (who likely have higher health literacy) ignored the survey.

Impact on Results: If the healthiest/highest literacy patients dropped out of the intervention group, the intervention effect might be underestimated. The authors MUST discuss this in the Limitations section (currently they only mention younger/healthier responded less, but don't connect the dots to health literacy bias). This is a major omission.

Discussion

"These findings suggest that there is no clear association... raising two possible explanations: either PASC did not directly affect health literacy domains, or the HLQ instrument lacked the sensitivity..."

This is the exact same sentence logic as the Abstract Conclusion. While consistency is good, the Discussion should expand on this, not repeat it verbatim. The first paragraph of a Discussion should summarize the main finding and then immediately pivot to interpretation.

Redundancy .please rewrite

The "Trend" Signal in Domain 2 is Ignored in the Discussion

Discussion mentions ceiling effects and qualitative findings but never addresses the near-significant 0.90 Beta in Domain 2.

[PASC is considered highly operational, context specific and oriented toward patient safety, and oriented toward patient safety

] “oriented toward patient safety” duplicated in one sentence delet one

Conclusion

The conclusion should synthesize the three key takeaways from the Discussion:

1. Primary Finding: No significant difference in HLQ scores.

2. Methodological Insight: Ceiling effects in this high-functioning Norwegian cohort likely masked any true effect; the HLQ may be inappropriate for this specific intervention evaluation.

3. Signal of Interest: The trend toward improved information management (Domain 2) aligns with qualitative feedback and warrants further investigation using more sensitive, context-specific tools.

**********

6. PLOS authors have the option to publish the peer review history of their article (what does this mean?). If published, this will include your full peer review and any attached files.

If you choose “no”, your identity will remain anonymous but your review may still be made public.

**Do you want your identity to be public for this peer review?**  For information about this choice, including consent withdrawal, please see our Privacy Policy.

Reviewer #1: No

Reviewer #2: No

**********

[NOTE: If reviewer comments were submitted as an attachment file, they will be attached to this email and accessible via the submission site. Please log into your account, locate the manuscript record, and check for the action link "View Attachments". If this link does not appear, there are no attachment files.]

To ensure your figures meet our technical requirements, please review our figure guidelines: https://journals.plos.org/plosone/s/figures

You may also use PLOS’s free figure tool, NAAS, to help you prepare publication quality figures: https://journals.plos.org/plosone/s/figures#loc-tools-for-figure-preparation.

NAAS will assess whether your figures meet our technical requirements by comparing each figure against our figure specifications.

# Author comment: Author response to Decision Letter 1

**DOI:** 10.1371/journal.pone.0352245.r002

30 May 2026

Dear Fatemeh Zarei, Academic Editor

Thank you very much for your rapid and through assessment of our manuscript: “Impact of a patient safety checklist on health literacy outcomes –A sub-study of a stepped wedge cluster randomized controlled trial “, and for the opportunity to submit a revised version. In what follows, we have restated the points raised by the editor/ reviewers (in italics) and detailed our response to each one of them- including the relevant amendments of the manuscript as required. The reviewer’s comments have been addressed chronologically.

Editors´comments:

When submitting your revision, we need you to address these additional requirements.

1. Please ensure that your manuscript meets PLOS ONE¨s style requirements, including those for file naming. The PLOS ONE style templates can be found at

https://journals.plos.org/plosone/s/file?id=wjVg/PLOSOne_formatting_sample_main_body.pdf and https://journals.plos.org/plosone/s/file?id=ba62/PLOSOne_formatting_sample_title_authors_affiliations.pdf

Response to Editor

Thank you for your guidance regarding the PLOS ONE style requirements. We have carefully reviewed our manuscript and made changes in accordance with the provided guidelines. All modifications have been clearly marked with track changes in addition to a clean manuscript.

2. We note that the grant information you provided in the “Funding Information” and “Financial Disclosure ‘sections do not match. When you resubmit, please ensure that you provide the correct grant numbers for the awards you received for your study in the “Funding Information” section.

If you would like to make changes to your financial disclosure, please include your updated statement in your cover letter.

Response to Editor

Thank you for your comments regarding the grant information. We have reviewed and corrected the discrepancies between the “Funding Information” and “Financial Disclosure” sections.

Additionally, we have updated our financial disclosure statement accordingly and have included the revised statement in the cover letter as instructed:

“Dr. Arvid Steinar Haugen applied for this study’s funding, and the Norwegian Council of Nurses awarded the project with funding for a doctoral scholarship grant (ID number: 2 – 2022). This funding was administered by the Department of Anaesthesia and Intensive Care, Haukeland University Hospital, Bergen, Norway, and corresponding author Ann Kristin Sandsbakk Austarheim (AKSA) was employed as the PhD candidate in the study. The co-authors Kristin Harris (KH), Hilde Valen Wæhle (HVW), Anette Storesund (AS), Randi Julie Tangvik (RJT), and Arvid Steinar Haugen (ASH) were affiliated with the larger Stepped Wedge Cluster Randomized Controlled Trial of PASC, which was funded by the Norwegian Council of Research (ID number:320475). This funding was administered by the Department of Anaesthesia and Intensive Care, Haukeland University Hospital, Bergen, Norway. The funders had no role in study design, data collection and analysis, decision to publish, or preparation of the manuscript, and only provided financial support in the form of authors' salaries and research materials.”

The URLs to sponsors of this study:

1. Norsk Sykepleierforbund - NSF

2. Implementation of Patients Safety Checklist (PASC) in Surgery, a Stepped Wedge Cluster RCT - Effects on Patient and Implementation Outcomes - Prosjektbanken

“The funders provided support in the form of salaries for authors [AKSA, KH, HVW, AS, RJT, and ASH], but did not have any additional role in the study design, data collection and analysis, decision to publish, or preparation of the manuscript. The specific roles of these authors are articulated in the ‘author contributions’ section.”

3. Thank you for stating the following in the Competing Interests section:

“We have read the journal's policy, and the authors of this manuscript have the following competing interests: Arvid Steinar Haugen is member of the European Society of Anaesthesiologists’ and Intensive Care (ESAIC) Patient Safety and Quality Committee, representing the International Federation of Nurse Anaesthetists (IFNA). The other authors have declared that no competing interests exist.”

Please confirm that this does not alter your adherence to all PLOS ONE policies on sharing data and materials, by including the following statement: "This does not alter our adherence to PLOS ONE policies on sharing data and materials.” (as detailed online in our guide for authors http://journals.plos.org/plosone/s/competing-interests). If there are restrictions on sharing of data and/or materials, please state these. Please note that we cannot proceed with consideration of your article until this information has been declared.

Please include your updated Competing Interests statement in your cover letter; we will change the online submission form on your behalf.

Response to Editor

The updated Competing Interests statement has been included in our cover letter as requested:

“We have read the journal's policy, and the authors of this manuscript have the following competing interests: Arvid Steinar Haugen is member of the European Society of Anaesthesiologists’ and Intensive Care (ESAIC) Patient Safety and Quality Committee, representing the International Federation of Nurse Anaesthetists (IFNA). The other authors have declared that no competing interests exist. This does not alter our adherence to PLOS ONE policies on sharing data and materials.”

4. Thank you for stating the following financial disclosure:

“The Norwegian Council of Nurses granted a doctoral scholarship to AKSA, through the Department of Anaesthesia and Intensive Care, Haukeland University Hospital, Bergen, Norway, for this study. Kristin Harris (KH), Hilde Valen Wæhle (HVW), Anette Storesund (AS), Randi Julie Tangvik (RJT), and Arvid Steinar Haugen (ASH) are affiliated with the Stepped Wedge Cluster Randomized Controlled Trial of PASC, which is funded by the Norwegian Council of Research (ID:320475). The funders had no role in study design, data collection and analysis, decision to publish, or preparation of the manuscript.”

We note that one or more of the authors is affiliated with the funding organization, indicating the funder may have had some role in the design, data collection, analysis or preparation of your manuscript for publication; in other words, the funder played an indirect role through the participation of the co-authors. If the funding organization did not play a role in the study design, data collection and analysis, decision to publish, or preparation of the manuscript and only provided financial support in the form of authors' salaries and/or research materials, please do the following:

1. Review your statements relating to the author contributions and ensure you have specifically and accurately indicated the role(s) that these authors had in your study. These amendments should be made in the online form.

Response to Editor:

We have in the online form made the following amendments:

“Dr. Arvid Steinar Haugen applied for this study’s funding, and the Norwegian Council of Nurses awarded the project with funding for a doctoral scholarship grant (ID number: 2 – 2022). This funding was administered by the Department of Anaesthesia and Intensive Care, Haukeland University Hospital, Bergen, Norway, and corresponding author Ann Kristin Sandsbakk Austarheim (AKSA) was employed as the PhD candidate in the study. The co-authors Kristin Harris (KH), Hilde Valen Wæhle (HVW), Anette Storesund (AS), Randi Julie Tangvik (RJT), and Arvid Steinar Haugen (ASH) were affiliated with the larger Stepped Wedge Cluster Randomized Controlled Trial of PASC, which was funded by the Norwegian Council of Research (ID number:320475). This funding was administered by the Department of Anaesthesia and Intensive Care, Haukeland University Hospital, Bergen, Norway. The funders had no role in study design, data collection and analysis, decision to publish, or preparation of the manuscript, and only provided financial support in the form of authors' salaries and research materials.”

The URLs to sponsors of this study:

1. Norsk Sykepleierforbund - NSF

2. Implementation of Patients Safety Checklist (PASC) in Surgery, a Stepped Wedge Cluster RCT - Effects on Patient and Implementation Outcomes - Prosjektbanken

2. Confirm in your cover letter that you agree with the following statement, and we will change the online submission form on your behalf:

“The funder provided support in the form of salaries for authors [insert relevant initials] but did not have any additional role in the study design, data collection and analysis, decision to publish, or preparation of the manuscript. The specific roles of these authors are articulated in the ‘author contributions’ section.”

Response to Editor:

We have confirmed in our cover letter that we agree with the proposed statement and that the changes can be made in the online submission form on our behalf. We appreciate your assistance in making the changes directly in the online submission form. We also note that since there are two funders, we have changed "funder" to "funders" accordingly.

“The funders provided support in the form of salaries for authors [AKSA, KH, HVW, AS, RJT, and ASH], but did not have any additional role in the study design, data collection and analysis, decision to publish, or preparation of the manuscript. The specific roles of these authors are articulated in the ‘author contributions’ section.”

5. In the online submission form you indicate that your data is not available for proprietary reasons and have provided a contact point for accessing this data. Please note that your current contact point is a co-author on this manuscript. According to our Data Policy, the contact point must not be an author on the manuscript and must be an institutional contact, ideally not an individual. Please revise your data statement to a non-author institutional point of contact, such as a data access or ethics committee, and send this to us via return email. Please also include contact information for the third-party organization, and please include the full citation of where the data can be found.

Response to Editor

Thank you for this comment. In response, we have revised the data availability statement as requested. The updated statement has been submitted in the online form and sent to you via return email. The revised statement reads as follows:

“Data is not publicly available for proprietary reasons due to restrictions on data sharing according to the ethics committee REK WEST (2016-1102). For researchers whose proposed use of the data has been approved for research or educational purposes, anonymous data can be accessed after approval of a proposal and a signed data access agreement. Requests must be made to the Department of Anaesthesia and Intensive Care, Haukeland University Hospital, e-mail: forskningksk@helse-bergen.no.”

6. If the reviewer comments include a recommendation to cite specific previously published works, please review and evaluate these publications to determine whether they are relevant and should be cited. There is no requirement to cite these works unless the editor has indicated otherwise.

Response to Editor

Thank you for this advice. No additional citations have been added based on reviewer suggestions.

Reviewers' comments:

Comments to the Authors

1-4 (checklist points yes/no).

Author’s responses to Reviewers

1-4. The authors have no responses to these check points.

5. Review Comments to the Authors

Reviewer #1: As the statistical reviewer I will focus on methods and reporting

Major

1) The study had power problems because many participants scored at the very top of the health literacy scales, leaving little room to detect improvement, and the observed statistical power across outcomes was very low, meaning the study was unlikely to detect real differences even if they existed. Although the authors mention ceiling effects and note this may limit the ability to detect changes, they do not explicitly acknowledge that the study was under-powered overall, nor do they directly link the non significant findings to inadequate power. A driver of this was the very large, assumed effect size.

Response to Reviewer#1

We thank the reviewer for highlighting the potential inadequate power of the study. As noted in the manuscript, a substantial proportion of participants scored at or near the upper limit of the health literacy scales, which may have reduced variability and limited the sensitivity to detect changes. While ceiling effects may have reduced the effective ability to detect change in this sample, we think this represents a measurement and sample characteristic limitation rather than inadequate a priori statistical power. We do acknowledge that the post hoc power observation indicated a low sample size and that variations in non-responder characteristics across the clusters, especially for non-responders could have influenced on the required sample size. Hence, we have included this possibility in the limitation section on page 17, lines 333-342:

“Several limitations should be considered when interpreting the results. The overall health literacy domain levels demonstrated ceiling effects in both control and intervention groups, which may have limited the ability to detect changes. Even though a priory sample size was estimated, it is possible that a higher number of respondents and participants could have increased the study’s overall ability to detect changes. Especially since younger and healthier participants responded less than older patients. In the general Norwegian population, younger people between 18-24 years are reported to have challenges with interacting with healthcare professionals [30]. Based on this underlying difference between responders and non-responders, we cannot exclude the risk that the statistical power was limited and could have increased with a larger sample.”

Reviewer#1

2) report uncertainty in Cronbach's alpha, e.g. bootstrap.

Response to Reviewer#1

Thank you for this comment. We found that the bootstrapping option for the Cronbach alphas were not available within the Reliability Analysis procedure in SPSS Statistics v. 31.0 However, we were able to produce equal confidence interval estimates by using the Intraclass correlation coefficient (ICC), absolute agreement, and reporting the average measures.

In Statistical Analysis we have added this information on page 9, lines 212-215:

“To account for variability in Cronbach’s alpha domain measures, we calculated 95% confidence intervals (CI’s) using the intraclass correlation coefficient (ICC) with absolute agreement, and the average measures.”

Under results, we have listed the results on page 14, lines 262-264, and added the 95% CI in the S3 Table:

“The Cronbach’s alpha values ranged from 0.725 to 0.873 across the nine domains and are reported with corresponding 95% CI’s in the S4 Table.”

Reviewer#1

3) no information as to how missing data were handled. all data were not complete I presume, so why weren't multiple imputation approaches used?

Response to Reviewer#1

Thank you very much for this comment. We agree that a more detailed description was warranted. We observed few missing values in the data set; the highest missing proportion was for patient characteristics as Education (n=20). And for the items in the nine domains, there were few missing values. The missing data was mainly completely at random (MCAR) for eight domains or random (MAR) for one domain. As the missing values did not exceed the original limits of the sample size calculations for our regression analysis, we did not apply any imputations for the missing values. However, based on the possibility for being under-powered we have now made new analysis, with multiple imputation in SPSS, both for Table 2 and Table 3. We have rephrased our text in Statistical Analyses, page 9 lines 203-211:

“Patterns of missing data were controlled for patient characteristics and the HLQ domain items. Baseline characteristics of responders and non responders were compared to assess potential attrition biases. Responders and non-responder differences were examined for the variables age

Attachment Submitted filename: Response to Reviewers.docx

# Peer review: Decision Letter 1

**DOI:** 10.1371/journal.pone.0352245.r003

**Authors:** Fatemeh Zarei, Fatemeh Zarei

7 Jun 2026

Effect of a Patient-Driven Perioperative Intervention on Health Literacy: A Stepped-Wedge Cluster Randomized Sub-Study

PONE-D-26-02760R1

Dear Dr. Ann Kristin Sandsbakk Austarheim

We’re pleased to inform you that your manuscript has been judged scientifically suitable for publication and will be formally accepted for publication once it meets all outstanding technical requirements.

Within one week, you’ll receive an e-mail detailing the required amendments. When these have been addressed, you’ll receive a formal acceptance letter and your manuscript will be scheduled for publication.

An invoice will be generated when your article is formally accepted. Please note, if your institution has a publishing partnership with PLOS and your article meets the relevant criteria, all or part of your publication costs will be covered. Please make sure your user information is up-to-date by logging into Editorial Manager at Editorial Manager® and clicking the ‘Update My Information' link at the top of the page. For questions related to billing, please contact billing support.

If your institution or institutions have a press office, please notify them about your upcoming paper to help maximize its impact. If they’ll be preparing press materials, please inform our press team as soon as possible -- no later than 48 hours after receiving the formal acceptance. Your manuscript will remain under strict press embargo until 2 pm Eastern Time on the date of publication. For more information, please contact onepress@plos.org.

Kind regards,

Fatemeh Zarei, PhD

Academic Editor

PLOS One

Additional Editor Comments (optional):

----

Reviewers' comments:

Reviewer's Responses to Questions

**Comments to the Author**

1. If the authors have adequately addressed your comments raised in a previous round of review and you feel that this manuscript is now acceptable for publication, you may indicate that here to bypass the “Comments to the Author” section, enter your conflict of interest statement in the “Confidential to Editor” section, and submit your "Accept" recommendation.

Reviewer #1: All comments have been addressed

Reviewer #2: All comments have been addressed

**********

2. Is the manuscript technically sound, and do the data support the conclusions?

The manuscript must describe a technically sound piece of scientific research with data that supports the conclusions. Experiments must have been conducted rigorously, with appropriate controls, replication, and sample sizes. The conclusions must be drawn appropriately based on the data presented.

Reviewer #1: Yes

Reviewer #2: Yes

**********

3. Has the statistical analysis been performed appropriately and rigorously?

Reviewer #1: Yes

Reviewer #2: Yes

**********

4. Have the authors made all data underlying the findings in their manuscript fully available?

The PLOS Data policy requires authors to make all data underlying the findings described in their manuscript fully available without restriction, with rare exception (please refer to the Data Availability Statement in the manuscript PDF file). The data should be provided as part of the manuscript or its supporting information, or deposited to a public repository. For example, in addition to summary statistics, the data points behind means, medians and variance measures should be available. If there are restrictions on publicly sharing data—e.g. participant privacy or use of data from a third party—those must be specified.

Reviewer #1: (No Response)

Reviewer #2: Yes

**********

5. Is the manuscript presented in an intelligible fashion and written in standard English?

PLOS ONE does not copyedit accepted manuscripts, so the language in submitted articles must be clear, correct, and unambiguous. Any typographical or grammatical errors should be corrected at revision, so please note any specific errors here.

Reviewer #1: Yes

Reviewer #2: (No Response)

**********

6. Review Comments to the Author

Please use the space provided to explain your answers to the questions above. You may also include additional comments for the author, including concerns about dual publication, research ethics, or publication ethics. (Please upload your review as an attachment if it exceeds 20,000 characters)

Reviewer #1: I am satisfied with the authors' responses and the resulting changes to the paper. I have nothing else to add.

Reviewer #2: Thanks for efforts . It was satisfying. All commnets point by points were adressed clearly

**********

7. PLOS authors have the option to publish the peer review history of their article (what does this mean?). If published, this will include your full peer review and any attached files.

If you choose “no”, your identity will remain anonymous but your review may still be made public.

**Do you want your identity to be public for this peer review?**  For information about this choice, including consent withdrawal, please see our Privacy Policy.

Reviewer #1: No

Reviewer #2: **Yes:**  Fatemeh Zarei

**********

# Editor report: Acceptance letter

**DOI:** 10.1371/journal.pone.0352245.r004

**Authors:** Fatemeh Zarei, Fatemeh Zarei

PONE-D-26-02760R1

PLOS One

Dear Dr. Austarheim,

I'm pleased to inform you that your manuscript has been deemed suitable for publication in PLOS One. Congratulations! Your manuscript is now being handed over to our production team.

At this stage, our production department will prepare your paper for publication. This includes ensuring the following:

* All references, tables, and figures are properly cited

* All relevant supporting information is included in the manuscript submission,

* There are no issues that prevent the paper from being properly typeset

You will receive further instructions from the production team, including instructions on how to review your proof when it is ready. Please keep in mind that we are working through a large volume of accepted articles, so please give us a few days to review your paper and let you know the next and final steps.

Lastly, if your institution or institutions have a press office, please let them know about your upcoming paper now to help maximize its impact. If they'll be preparing press materials, please inform our press team within the next 48 hours. Your manuscript will remain under strict press embargo until 2 pm Eastern Time on the date of publication. For more information, please contact onepress@plos.org.

You will receive an invoice from PLOS for your publication fee after your manuscript has reached the completed accept phase. If you receive an email requesting payment before acceptance or for any other service, this may be a phishing scheme. Learn how to identify phishing emails and protect your accounts at https://explore.plos.org/phishing.

If we can help with anything else, please email us at customercare@plos.org.

Thank you for submitting your work to PLOS ONE and supporting open access.

Kind regards,

PLOS ONE Editorial Office Staff

on behalf of

Dr. Fatemeh Zarei

Academic Editor

PLOS One
